# Integrative genomic analysis and diagnostic modeling of osteoporosis: unraveling the interplay of autophagy, osteogenesis, adipogenesis, and immune infiltration

**DOI:** 10.3389/fmed.2025.1544390

**Published:** 2025-04-17

**Authors:** Lin-Jing Han, Jian-Zong Zhu, Hong-Cai Liu, Xiao-Sheng Lin, Shu-Zhong Yang

**Affiliations:** ^1^Orthopedics Department, Shenzhen Hospital of Integrated Traditional Chinese and Western Medicine, Shenzhen, China; ^2^Graduate School, Guangzhou University of Chinese Medicine, Guangzhou, China; ^3^Shenzhen Bao’an Chinese Medicine Hospital, Guangzhou University of Chinese Medicine, Shenzhen, China; ^4^Osteoporosis Department, Baoan Central Hospital of Shenzhen, Shenzhen, China

**Keywords:** osteoporosis, bioinformatics, autophagyosteogenesis, immune infiltration, diagnostic biomarkers

## Abstract

**Background:**

Osteoporosis (OP), marked by reduced bone density and structural decay, poses a heightened risk of fractures. Our study formulates a predictive diagnostic model for OP by analyzing differential gene expression, thereby improving early diagnosis and therapeutic approaches.

**Methods:**

Using GSE62402, GSE56815, and GSE35958 datasets from the Gene Expression Omnibus (GEO) database, we identified differentially expressed genes (DEGs) via R packages, and evaluated the underlying molecular mechanisms by network analysis. Immune checkpoint and drug sensitivity were analyzed to construct and validate diagnostic models. The single-sample gene-set enrichment analysis (ssGSEA) was used to assess immune cell infiltration; the CIBERSORT algorithm was used to evaluate immune cells within the different subtypes of OP.

**Results:**

The study identified 1,297 DEGs, with 14 DEGs related to autophagy, osteogenesis, and adipogenesis (AP&OG&AGRDEGs) showing significant expression differences between OP and control groups, including seven upregulated and seven downregulated genes (*p*-value < 0.05). The analysis results from gene ontology (GO), gene set enrichment analysis (GSEA), and the Kyoto encyclopedia of genes and genomes (KEGG) indicated that oxidative stress and inflammation-related signaling pathways are closely connected to OP. Immune checkpoint analysis identified differential expression of eight genes between OP patients and controls (*p*-value < 0.05). The ssGSEA findings showed significant variations in immune cell infiltration levels, particularly of natural killer cells, Th2 cells, mast cells, and plasmacytoid dendritic cells (*p*-value < 0.05). The diagnostic model, developed utilizing logistic regression, support vector machine (SVM), and the least absolute shrinkage and selection operator (LASSO), pinpointed nine pivotal genes—*AKT1, NFKB1, TNF, CTNNB1, LMNA, BHLHE40, BMP4, WNT1, and COPS3*—and confirmed their diagnostic efficacy through validation. In further subgroup analysis, eight types of immune cells were found to be differentially expressed across various risk groups. Subtype analysis based on ConsensusClusterPlus revealed differential expression of six key genes in distinct subtypes of OP.

**Conclusion:**

This comprehensive study established a network of OP-associated genes, and provides insights into the molecular mechanisms involving immune responses in OP. It identified key diagnostic genes and analyzed immune cell infiltration to better understand OP pathogenesis. The study underscores the importance of personalized treatment and the potential role of immune modulation in managing OP.

## Introduction

Osteoporosis (OP) is a systemic skeletal disorder characterized by reduced bone density and the deterioration of bone microstructure, resulting in increased bone vulnerability and an elevated propensity for fractures (1). Dual-energy X-ray absorptiometry (DXA) remains the gold standard for diagnosis; however, DXA primarily detects changes in bone mineral density (BMD), exhibits limited predictive power for early-stage OP, and cannot provide molecular-level information. Annually, there are approximately 8.9 million osteoporotic fractures worldwide (2). Mortality rates for osteoporotic hip fractures vary widely over 5 years post-fracture, ranging from 38 to 64% depending on age, and ranging from 29 to 50% for vertebral fractures (3). A variety of therapeutic interventions have been developed for treating OP, including bisphosphonates, estrogen therapy to selective estrogen receptor modulators (SERMs), calcitonin, denosumab, and teriparatide (4). Though current treatments can improve bone loss, long - term use may cause adverse reactions. Thus, there’s an urgent need for novel molecular markers and diagnostic models to boost the accuracy of early diagnosis and supply a basis for personalized treatment.

As an insidious chronic disease, OP is influenced by numerous genetic and extrinsic factors, including autophagy activation (5), chronic inflammation (6), oxidative stress (7), autoimmunity (8), and bone metabolism imbalance. Enhancing autophagy within bone marrow mesenchymal stem cells (BMSCs) could promote bone formation. In older individuals, the osteogenic potential of BMSCs diminishes, while their propensity for adipogenic differentiation increases (9). Additionally, older individuals’ BMSCs exhibit significantly reduced autophagy levels compared to younger individuals, with the potential for autophagy activation to mitigate age-induced cellular decline (10). Based on previous findings, immune checkpoint molecules play a role in skeletal system inflammation resulting in OP. Chronic low-grade inflammation state could be influenced by estrogen deficiency (11). In ovariectomized mice (OVX), the proliferation of TNF-producing T cells increases the bone marrow (12). *RANKL*, a member of the *TNF* superfamily, facilitates the genesis and functionality of osteoclast (OC) through its binding to the *RANK* receptor present on OC progenitor cells (13). Moreover, the immune checkpoint molecule programmed cell death protein 1 (PD-1) could enhance osteoclastic activity and promote osteoclastogenesis, while programmed cell death 1 ligand 2 (PD-L2) could reduce the number of OC (14). Overall, current studies on OP molecular mechanisms predominantly focus on isolated pathways. However, integrative analyses bridging autophagy, osteogenesis, adipogenesis, and immune infiltration remain scarce. Our study pioneers a multi-omics approach to construct a molecular network encompassing these four dimensions, offering novel insights into OP diagnosis and immunomodulatory therapies.

In the past few years, using previous data, a vast number of genetic alterations have been screened at the genome level using microarray technology and bioinformatic analysis (15). Using microarray profiling, signaling pathways, biological processes, and promising targets have been identified for anti-OP. Recently, several microarray and bioinformatics studies have significantly advanced our understanding of the molecular events underlying OP pathogenesis (16, 17). Based on three datasets, 52 OP vs. 57 non-OP patients, a bioinformatics study identified 10 OP hub genes and six diagnostic genes; the results were verified in OP patients (18). However, the small RNA-related regulatory network remains underexplored. Ferroptosis, closely related to inflammation and immunity, has also been implicated in OP (19). In another study, with five OP vs. four non-OP patients data, bioinformatics identified five genes that are highly correlated with ferroptosis and OP; the result was validated in OVX mice (16). However, immune checkpoint analysis was included, and the small sample sizes and missing genotypic data may have led to false positives and reduced reliability of the results. There are few bioinformatics research data on the relationship between autophagy and OP.

Consequently, our study aims to integrate data from three mRNA microarray datasets retrieved from the Gene Expression Omnibus (GEO) database, focusing on identifying genes with substantial differential expression between blood samples from OP patients and healthy individuals. This step is fundamental for pinpointing the genetic signatures of OP. To further dissect the intricate molecular landscape, network analysis is essential for revealing the complex interactions and potential mechanisms driving OP progression, particularly the interplay between autophagy, osteogenic, and adipogenic differentiation. Building on these insights, we proceed with immune checkpoint and drug sensitivity analysis to identify therapeutic targets. Concurrently, we constructed and validated a diagnostic model to enhance early detection. By constructing small RNA regulatory networks and performing immune infiltration analysis, we aimed to classify OP subtypes, facilitating progress toward personalized medicine. Our research has established a comprehensive network of OP that encompasses autophagy, osteogenesis, and adipogenesis. This framework is a significant step forward in understanding the molecular mechanisms of OP and advancing its diagnosis at the systems biology level.

## Materials and methods

### Data download

The datasets pertinent to OP, namely GSE62402, GSE56815 (20), and GSE 35958 (21), were extracted from the GEO database utilizing the R package GEOquery (22). For more information, refer to Supplementary Table S1. In the datasets, GSE62402 has five samples with OP and five samples without; GSE56815 has 40 OP samples and 40 without; and GSE35958 has another five OP samples and four without. GSE35958 was selected as an independent validation set due to its complementary sample source (BMSCs) and phenotypic consistency (OP vs. non-OP) with the combined datasets (peripheral blood). Despite its small sample size (5 OP vs. 4 controls), its platform compatibility (GPL570) and probe standardization ensured validation reliability. All OP and control samples were included in this study.

Autophagy-related genes (APRGs), osteogenic-related genes (OGRGs), and adipogenic-related genes (AGRGs) were retrieved from the GeneCards database (23). Upon querying ‘Autophagy’, we identified genes as ‘Protein Coding’ with a ‘Relevance Score’ threshold of 0.5 or higher, resulting in the identification of a total of 5,152 APRGs. Additionally, by employing ‘Autophagy’ as a search term on the PubMed database (24), a collection of 223 published APRGs was procured. Upon consolidation and deduplication, a total of 5,177 APRGs were pinpointed, with detailed information presented in Supplementary Table S2. Subsequently, keyword searches for ‘Osteogenic’ and ‘Adipogenic’ identified 971 OGRGs and 292 AGRGs, respectively, with their specific characteristics outlined in Supplementary Tables S3, S4.

The datasets GSE62402 and GSE56815 were processed to remove batch effects using the R package sva (25), resulting in the formation of Combined Datasets (CDs). This dataset comprises 45 OP samples and an equal number of control samples. Subsequently, the R package limma (26) was employed for the normalization of the CDs, ensuring the standardization of annotation probes. The dataset GSE35958 served as a validation set following the probe annotation process.

### OP-related differentially expressed genes

Within the CDs, differential gene expression was analyzed for the OP and control groups with the limma R package (26). DEGs were filtered by |logFC| > 0 and *p*-value < 0.05. Upregulation was denoted by logFC > 0 and p-value < 0.05, and downregulation by logFC < 0 and the same p-value threshold. The volcano plot, created with ggplot2, and the heatmap from pheatmap, visualized the DEGs and gene expression profiles, respectively. To obtain the AP&OG&AGRDEGs, an intersection was taken between all the DEGs identified from the CDs and the autophagy, osteogenic, adipogenic-related genes (AP&OG&AGRGs). This intersection was then visualized using a Venn diagram. The resulting genes were further characterized by their chromosomal locations, which were depicted by the R package RCircos (27). This visualization provided a genomic mapping of the AP&OG&AGRDEGs associated with OP.

### Gene ontology and Kyoto encyclopedia of genes and genomes enrichment analysis

GO analysis, a prevalent tool for extensive functional enrichment assesses biological process (BP), cellular component (CC), and molecular function (MF) (28). The KEGG database is extensively utilized for its repository of genomic, pathway, disease, and drug data (29). Employing clusterProfiler (30) in R, we executed GO and KEGG enrichment analyses on the AP&OG&AGRDEGs, with statistical significance determined by an adjusted *p*-value < 0.05 and an FDR-value (q-value) < 0.25, using the Benjamini-Hochberg (BH) approach for p-value correction.

### Gene set enrichment analysis

GSEA (31) assesses the collective influence of pre-defined gene sets on a phenotype by examining the distribution patterns of genes within a ranked list that correlates with the phenotype. In our research, the initial step involved segmenting the CDs into distinct OP and control cohorts. Subsequently, we deployed the R package clusterProfiler to execute GSEA for the entire gene complement of the CDs, leveraging the logFC values. The GSEA parameters were configured with a seed value of 2020, a minimum gene set size threshold at 10, and a maximum gene set size capped at 500. This GSEA utilized gene sets derived from the Molecular Signatures database (MSigDB) (32), specifically the c2.cp.all.v2022.1.Hs.symbols.gmt [all canonical pathways] (3050) gene sets. The selection criteria were *p*-value < 0.05 and *q*-value < 0.25.

### Immune checkpoint and drug sensitivity analysis

Immune checkpoint genes (ICGs) are ligand-receptor pairs that could either inhibit or stimulate immune responses. They are vital in modulating the immune system to preserve balance and prevent autoimmune diseases. We identified 47 ICGs from published literature on the PubMed website (33), with specific gene names listed in Supplementary Table S5. We compared ICG expressions between the OP and control groups in the CDs, producing group comparison charts.

The Genomics of Drug Sensitivity in Cancer (GDSC) (34) database is a comprehensive public repository for data on cancer cell drug sensitivity and drug response molecular markers. Employing the pRRophetic algorithm (35), we predicted the sensitivity of OP patients to various anticancer drugs and small molecules using the expression profiles from the CDs to estimate IC50 values and visualized these findings in comparative charts.

### Immune infiltration analysis of OP

Utilizing single-sample gene-set enrichment analysis (ssGSEA) (36), we quantified the infiltration abundance of distinct immune cell populations, including specific subtypes like activated CD8 + T cells and dendritic cells. The ssGSEA scores were indicative of the relative presence of these cells within the samples. We applied a stringent *p*-value filter of less than 0.05 to refine the dataset into an immune infiltration matrix. The comparative analysis revealed disparities in immune cell abundance between the OP group and control group across the CDs, as evidenced by the group charts. Finally, heatmaps were crafted using the R package pheatmap to visually represent the intricate relationships among immune cells and their associations with pivotal genes. Finally, scatter plots were constructed to represent the correlations with immune cells that exhibited the highest positive and negative associations with key genes.

### Establishment of OP diagnostic model

For the construction of an OP diagnostic model using the CDs, logistic regression was applied to the AP&OG&AGRDEGs. Logistic regression is the method of choice for examining the relationship between independent variables and a binary dependent variable, like the presence or absence of OP, with statistical significance set at a *p*-value < 0.05. The molecular expressions of the AP&OG&AGRDEGs that were integrated into the logistic regression model were subsequently depicted in a forest plot. Subsequently, leveraging the AP&OG&AGRDEGs identified in the logistic regression model, a support vector machine (SVM) model was formulated employing the SVM algorithm (37). The AP&OG&AGRDEGs were selected for their optimal balance between accuracy and error rate. Subsequently, a least absolute shrinkage and selection operator (LASSO) regression analysis was conducted on the AP&OG&AGRDEGs from the SVM model, utilizing the R package glmnet (38) with a seed parameter set to 500. LASSO regression, an extension of linear regression, incorporates a penalty term (lambda times the absolute value of the slope coefficient) to mitigate overfitting, thereby enhancing the model’s predictive accuracy and generalizability. The outcome of the LASSO regression analysis is an OP diagnostic model with the AP&OG&AGRDEGs as pivotal genes. The findings were graphically represented through diagnostic and variable trajectory plots.

### Validation of OP diagnostic model

A nomogram (39) is a visual tool that represents the correlation between multiple variables with a series of parallel lines on a Cartesian coordinate plane. Utilizing the outcomes from the LASSO logistic regression analysis, the rms R package was employed to create a nomogram that maps the interactions among the key genes. A calibration curve was generated through calibration analysis to assess the precision and discriminatory power of the OP diagnostic model predicated on these key genes. Employing the ggDCA R package, a decision curve analysis (DCA) (40) was conducted to plot the DCA graph based on the key genes identified. DCA offers a clear method for assessing the clinical value of predictive models, diagnostic tests, and molecular biomarkers. Following this, the pROC R package was utilized to generate the receiver operating characteristic (ROC) curve for the risk score and to compute the area under the curve (AUC), which serves to evaluate the diagnostic performance of the risk score’s gene expression levels for predicting OP. The computation of the risk score adheres to the following formula:


$$\mathrm{risk}\mathrm{Score}=\underset{i}{\Sigma }\mathrm{Coefficient}\;\left(gene_{i}\right)*\mathrm{mRNA}\;\mathrm{Expression}\;\left(gene_{i}\right)$$

Furthermore, the OP samples were stratified into high- and low-risk groups using the median RiskScore value. To delve deeper into the expression variances of the key genes within the high- and low-risk OP samples, comparative charts were crafted reflecting the expression profiles of these genes. Ultimately, the pROC R package was employed to trace the ROC curves and to compute the AUC, thereby assessing the diagnostic accuracy of the key gene expression levels in predicting the development of OP.

### Profiling immune infiltration in high- and low-risk groups

With ssGSEA, we quantified the variations in immune cell infiltration among high- versus low-risk OP samples, filtering significant samples at a *p*-value < 0.05 for comparative visualization. The pheatmap tool in R was subsequently applied to illustrate the correlational patterns between immune cells and their relationships with key genes across the risk-stratified OP groups. Ultimately, immune cells that had the highest positive and negative correlations with key genes were identified, and scatter plots were constructed to depict these correlations.

### Key gene expression validation and correlation assessment

To delve deeper into the expression variances of key genes across the CDs and the validation set GSE35958, comparative expression charts were crafted for the OP and control groups in both datasets. Subsequently, the Spearman method was utilized to assess correlations among key gene expressions within the CDs and the GSE35958 dataset. The correlation heatmap, created with the R package pheatmap, visualized these findings.

### Construction of regulatory network

MicroRNAs (miRNAs) are instrumental in the modulation of biological systems and genetic adaptation, targeting a diverse set of genes. The phenomenon of a single gene being regulated by multiple miRNAs is also prevalent. To decipher the intricate links between miRNAs and key genes, we conducted an analysis using the TarBase (41) database to identify miRNAs that are associated with key genes. Additionally, transcription factors (TFs) regulate gene expression by binding to key genes post-transcriptionally. Thereafter, the influence of these transcription factors on key genes was examined by utilizing data obtained from the ChIPBase database (42). Key gene-associated drug targets, both direct and indirect, were predicted through the Comparative Toxicogenomics Database (CTD) (43) to assess the connectivity between key genes and pharmaceuticals. The subsequent visualization of the regulatory networks, including mRNA-miRNA, mRNA-TF, and mRNA-Drug relationships, was accomplished with Cytoscape software.

### OP subtypes construction

The algorithm of consensus clustering (44), which uses resampling to classify members into subgroups and to check the clusters’ coherence, was implemented via the R package ConsensusClusterPlus (45) to classify OP into its specific subtypes through the analysis of key genes. During this process, the cluster count was configured to vary between two and nine, executing 50 iterations with 80% of the sample size drawn each time, using “kmeans” for the clustering algorithm and “pearson” as the distance metric in the analysis. Subsequently, heatmaps were generated to delineate the key gene expression variances among OP’s subtypes. Furthermore, comparative group charts were implemented to affirm the gene expression disparities. In conclusion, the pheatmap R package was leveraged to exhibit the key genes’ correlation profiles within OP samples.

Immune infiltration analysis of subtypes of OP.

The ssGSEA quantifies global immune cell abundance via single-sample gene set enrichment, while CIBERSORT (46) deconvolutes specific immune subsets using linear support vector regression. This dual approach comprehensively characterizes OP immune microenvironment heterogeneity and thus aid in assessing immune cell distribution and proportions in mixed cellular environments. In our research, the CIBERSORT algorithm was implemented, utilizing the LM22 gene signature matrix, and we filtered the data to include only those instances where the immune cell enrichment scores exceeded zero, culminating in the derivation of a detailed immune cell infiltration matrix. Subsequently, the pheatmap R package was used to demonstrate the correlation analysis between the LM22 immune cell signatures and the key genes, specifically within the contexts of Cluster1 and Cluster2.

### Statistical analysis

Utilizing R software (Version 4.2.2), this article’s data processing and analysis presented continuous variables as mean ± standard deviation. For comparing any two groups, the Wilcoxon rank sum test was implemented. Spearman correlation analysis was the method for determining the correlation coefficients among various molecules, with a *p*-value threshold of less than 0.05 to denote statistical significance.

## Results

### Technology roadmap

**Figure 1:**
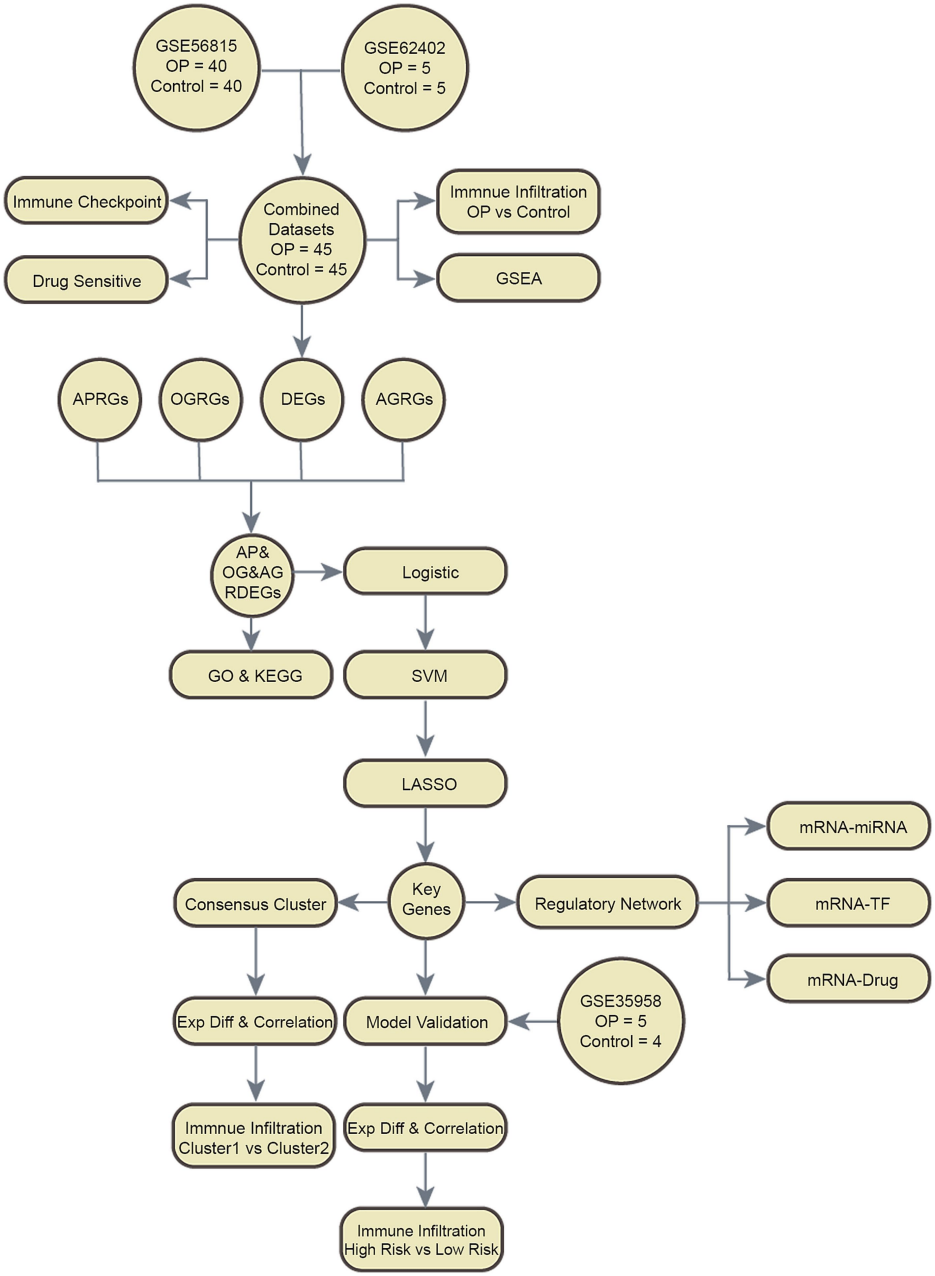
Flow chart for the comprehensive analysis of AP&OG&AGRDEGs. OP, osteoporosis; DEGs, differentially expressed genes; APRGs, autophagy-related genes; OGRGs, osteogenic-related genes; AGRGs, adipogenic-related genes; GSEA, gene set enrichment analysis; GO, gene ontology; KEGG, Kyoto Encyclopedia of Genes and Genomes; AP&OG&AGRDEGs, autophagy&osteogenic&adipogenic-related differentially expressed genes; SVM, support vector machine; LASSO, least absolute shrinkage and selection operator; ExpDiff, expression difference; TF, transcription factor.

The overarching structure of our research design is elegantly captured in Figure 1, which delineates the sequence of methodologies and analyses employed throughout the study.

### Pooling of OP datasets

**Figure 2:**
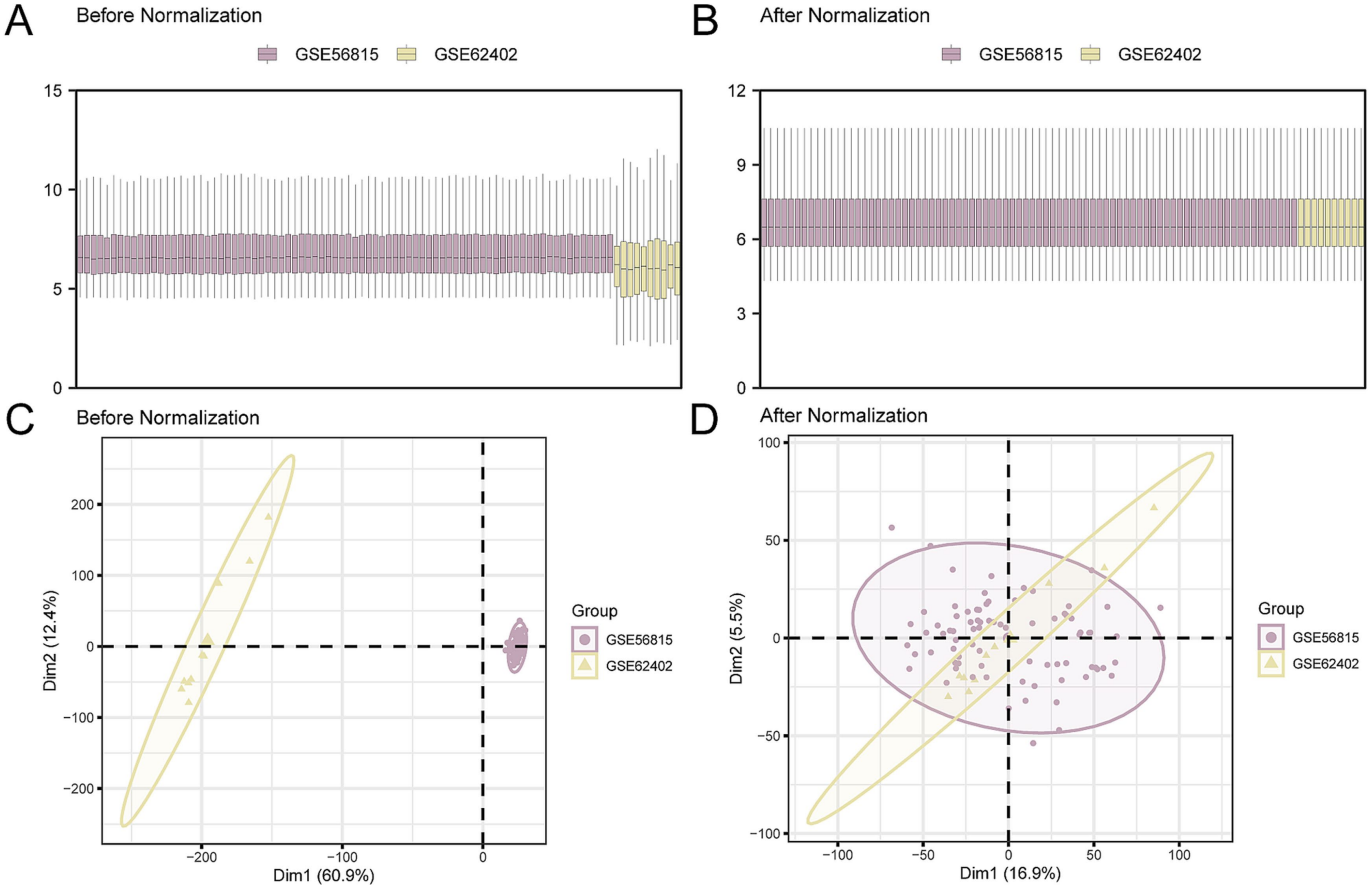
Batch effects removal of GSE56815 and GSE62402. **(A,B)**. Boxplot of the distribution of the CDs before **(A)** and after **(B)** de-batching. **(C,D)**. PCA plots of the CDs before **(C)** and after **(D)** de-batching. PCA, Principal Component Analysis; OP, osteoporosis; CDs, combined datasets. Purple is OP dataset GSE56815 and yellow is OP dataset GSE62402.

In our study, batch effects in the OP datasets GSE56815 and GSE62402 were mitigated using the ‘sva’ R package. After the batch effect correction, we consolidated the datasets into CDs. Subsequently, to visually assess the datasets before and after the batch effect removal, we employed distribution box plots and principal component analysis (PCA), as depicted in Figures 2A–D. The findings indicate that the batch effects within the CDs were largely neutralized.

### DEGs related to autophagy, osteogenesis, and adipogenesis

**Figure 3:**
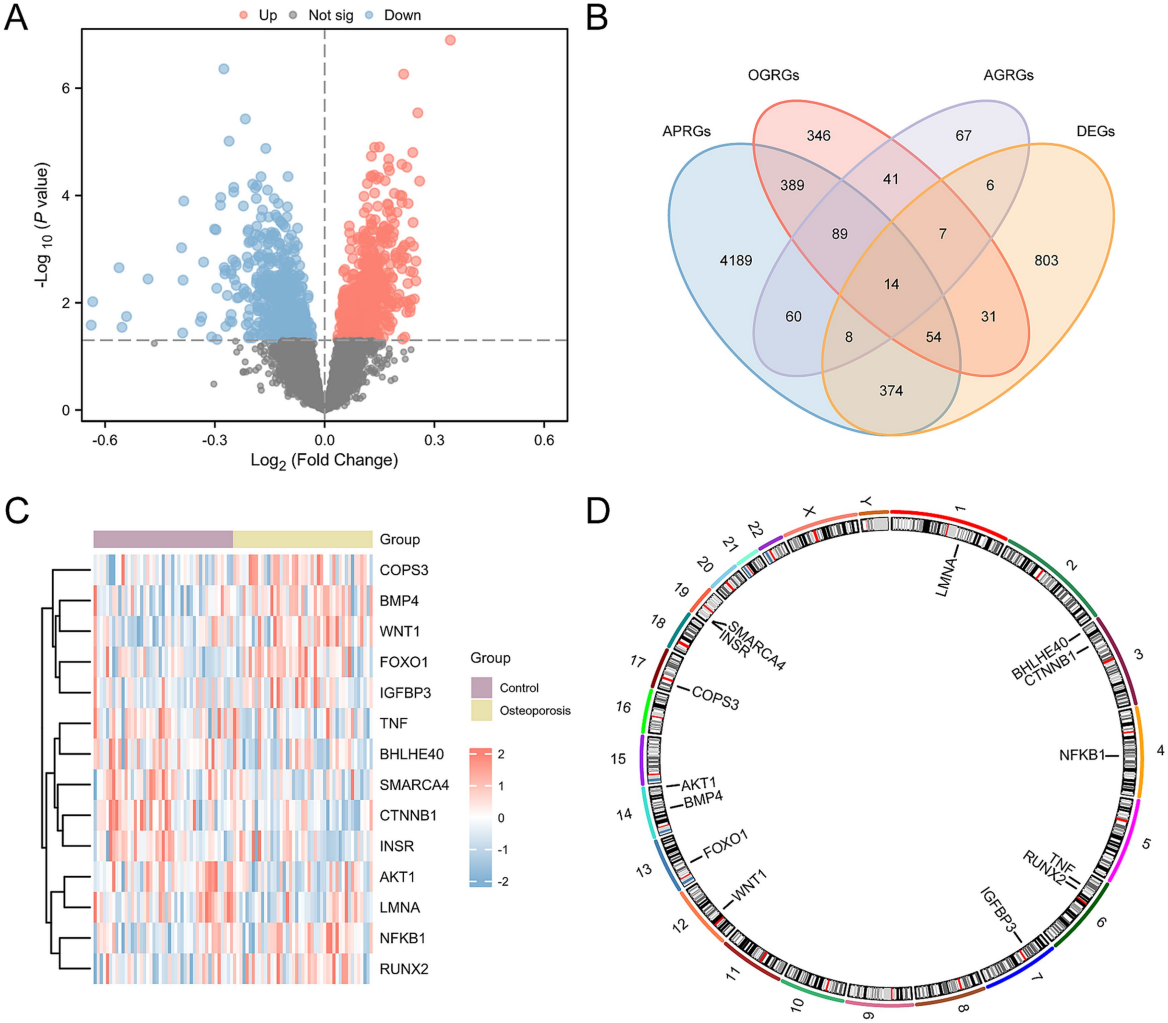
Differential gene expression analysis for CDs. **(A)** Volcano plots of differentially expressed genes in the OP and control groups in the CDs. **(B)** Venn plots of DEGs and AP&OG&AGRGs. **(C)** Heatmap of AP&OG&AGRDEGs in the CDs. **(D)** Chromosomal mapping of AP&OG&AGRDEGs. OP, osteoporosis; DEGs, differentially expressed genes; AP&OG&AGRGs, Autophagy&Osteogenic&Adipogenic-Related genes; AP&OG&AGRDEGs, Autophagy&Osteogenic&Adipogenic-related differentially expressed genes; CDs, combined datasets. Purple represents the control group, and yellow is the OP group.

To analyze the gene expression differences between OP and the control group, the ‘limma’ R package was used to conduct differential analysis on the CDs. In the ensuing analysis, all 1,297 DEGs were identified across the CDs that met the criteria of an absolute log fold change (|logFC|) > 0.00 and *p*-value < 0.05. Among these DEGs, 689 genes were upregulated, characterized by a logFC > 0.00 and a p-value < 0.05, and 608 genes were downregulated (logFC < 0.00 and p-value < 0.05). The results of the differential analysis were presented using a volcano plot (Figure 3A).

To identify AP&OG&AGRDEGs, we intersected the set of all DEGs above with the set of genes related to autophagy, osteogenesis, and adipogenesis (AP&OG&AGRGs). A total of 14 AP&OG&AGRDEGs were obtained and visualized using a Venn diagram (Figure 3B), including: *AKT1*, *NFKB1*, *FOXO1*, *TNF*, *CTNNB1*, *LMNA*, *INSR*, *BHLHE40*, *RUNX2*, *IGFBP3*, *BMP4*, *SMARCA4*, *WNT1*, and *COPS3*. Subsequently, the expression differences of these 14 AP&OG&AGRDEGs were analyzed and displayed by a heatmap (Figure 3C). Finally, the positions of the 14 AP&OG&AGRDEGs on the human chromosomes were mapped using the R package RCircos, and a chromosomal location map was created (Figure 3D). The results indicate that the majority of the AP&OG&AGRDEGs are located on chromosomes 3, 6, 14, and 19.

### GO and KEGG enrichment analysis

**Figure 4:**
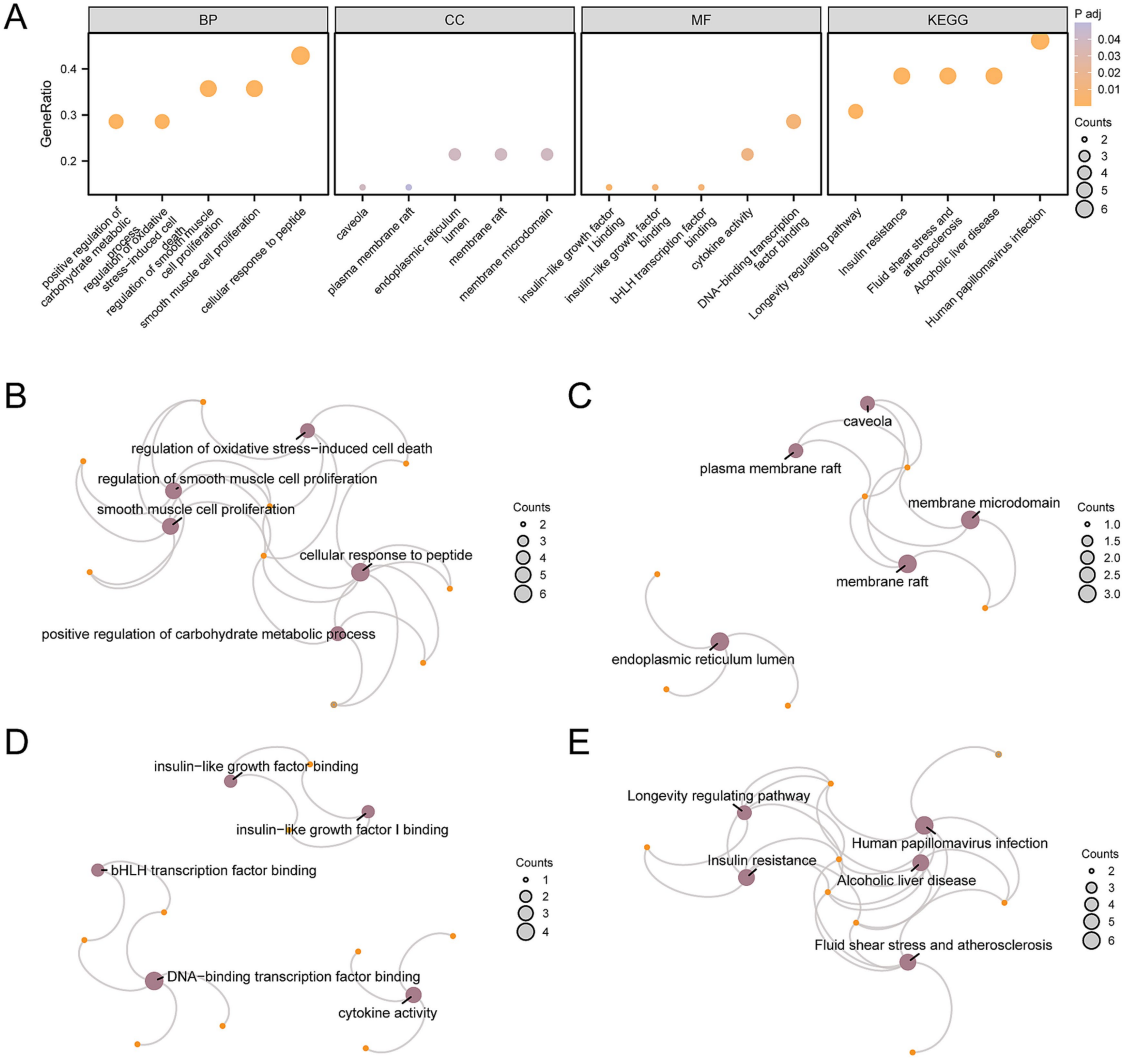
GO and KEGG enrichment analysis for AP&OG&AGRDEGs.**(A)** Bubble diagram of GO and KEGG enrichment analysis results of AP&OG&AGRDEGs: BP, CC, MF, and biological pathways. The abscissa is GO terms and KEGG terms. **(B–E)** Network diagram of GO and KEGG enrichment analysis results of AP&OG&AGRDEGs: BP **(B)**, CC **(C)**, MF **(D)** and KEGG **(E)**. Purple nodes represent items, orange nodes represent molecules, and connecting lines represent the relationship between items and molecules. AP&OG&AGRDEGs, Autophagy&Osteogenic&Adipogenic-related differentially expressed genes; GO, gene ontology; KEGG, Kyoto Encyclopedia of Genes and Genomes; BP, biological process; CC, cellular component; MF, molecular function. The item screening criteria for GO and KEGG enrichment analyses were adj. *p*-value < 0.05 and FDR-value (*q*-value) < 0.25 were considered statistically significant, with Benjamini-Hochberg (BH) as the *p*-value correction method.

To analyze the biological mechanisms involved with these 14 AP&OG&AGRDEGs, we conducted GO and KEGG enrichment analysis, with detailed results presented in Supplementary Table S6. The analyses revealed that the AP&OG&AGRDEGs were significantly enriched in BP, including the regulation of smooth muscle cell proliferation, the regulation of cell death induced by oxidative stress, and the proliferation of smooth muscle cells. In terms of CC, they were enriched in the endoplasmic reticulum lumen, membrane raft, and membrane microdomain. For MF, they were involved in binding insulin-like growth factor I, insulin-like growth factor, and bHLH transcription factor. Additionally, they were enriched in biological pathways including insulin resistance, fluid shear stress and atherosclerosis, alcoholic liver disease, human papillomavirus infection, and longevity regulating pathways. The outcomes of the GO and KEGG enrichment analyses were depicted through a bubble chart (Figure 4A), with the top 5 BP/CC/MF terms and pathways shown in Supplementary Table S6. Simultaneously, a network diagram was generated to illustrate the BP, CC, MF, and KEGG pathways as determined by the enrichment analysis (Figures 4B–E).

### GSEA for CDs

**Figure 5:**
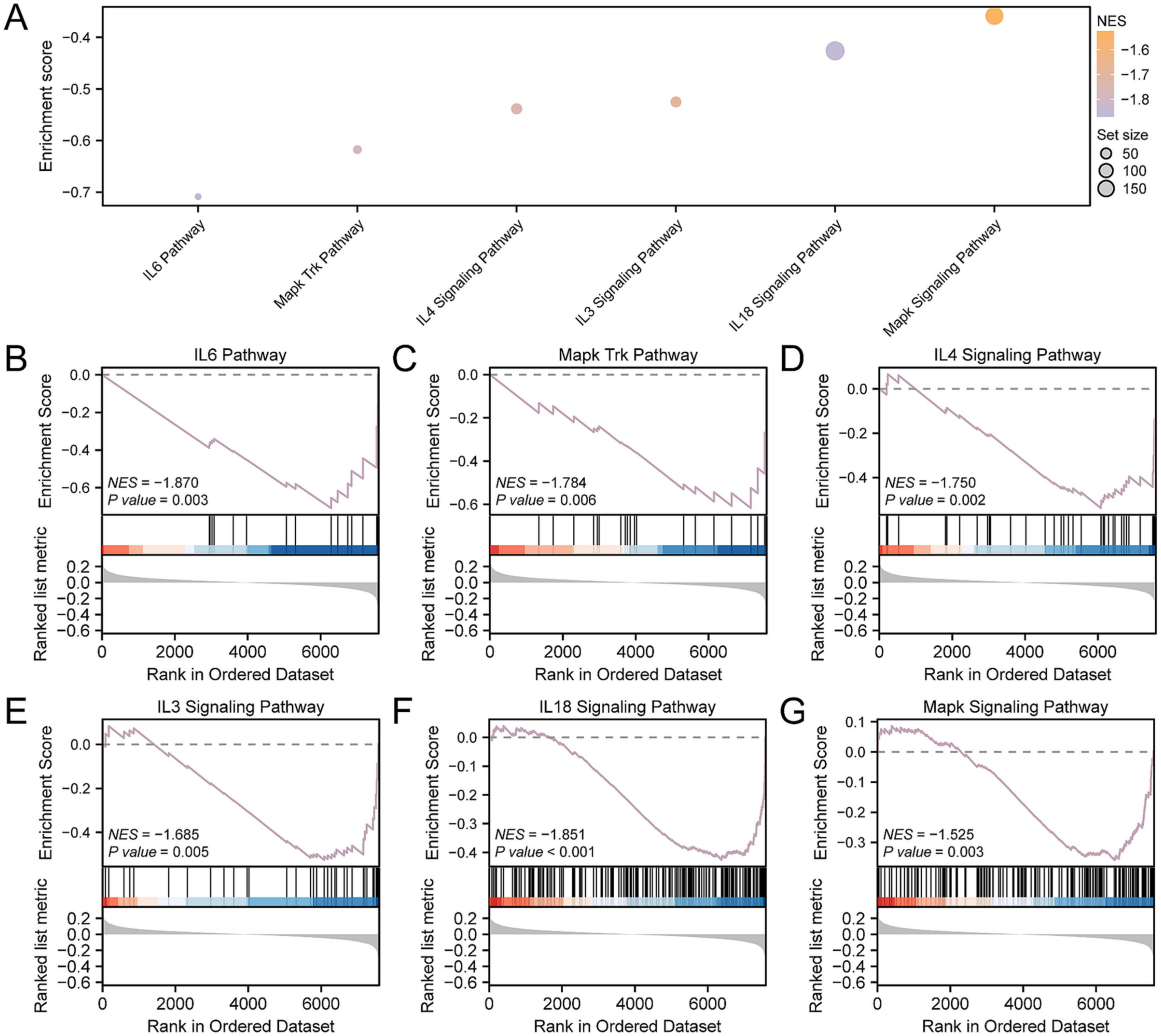
GSEA for CDs. **(A)** GSEA of the CDs is presented in a mountain map of six biological functions. **(B–G)** GSEA showed that OP was significantly enriched in IL6 **(B)**, Mapk Trk **(C)**, IL4 **(D)**, IL3 **(E)**, IL18 **(F)** and Mapk signaling pathways **(G)**. OP, osteoporosis; GSEA, gene set enrichment analysis; CDs, combined datasets. GSEA screening criteria were *p*- value < 0.05 and FDR-value (*q*-value) < 0.25.

To ascertain the influence of gene expression levels across the CDs on OP, GSEA was utilized to examine the interplay between gene expression and the associated biological processes, impacted cellular components, and engaged molecular functions (Figure 5A). Further details of the results are available in Supplementary Table S7. The findings indicated that the genes within the CDs were substantially enriched across a spectrum of biological functions and signaling pathways, including the IL6 (Figure 5B), MAPK-Trk (Figure 5C), IL4 (Figure 5D), IL3 (Figure 5E), IL18 (Figure 5F), and the overarching MAPK signaling pathways (Figure 5G).

### Immune checkpoint and drug sensitivity analysis

**Figure 6:**
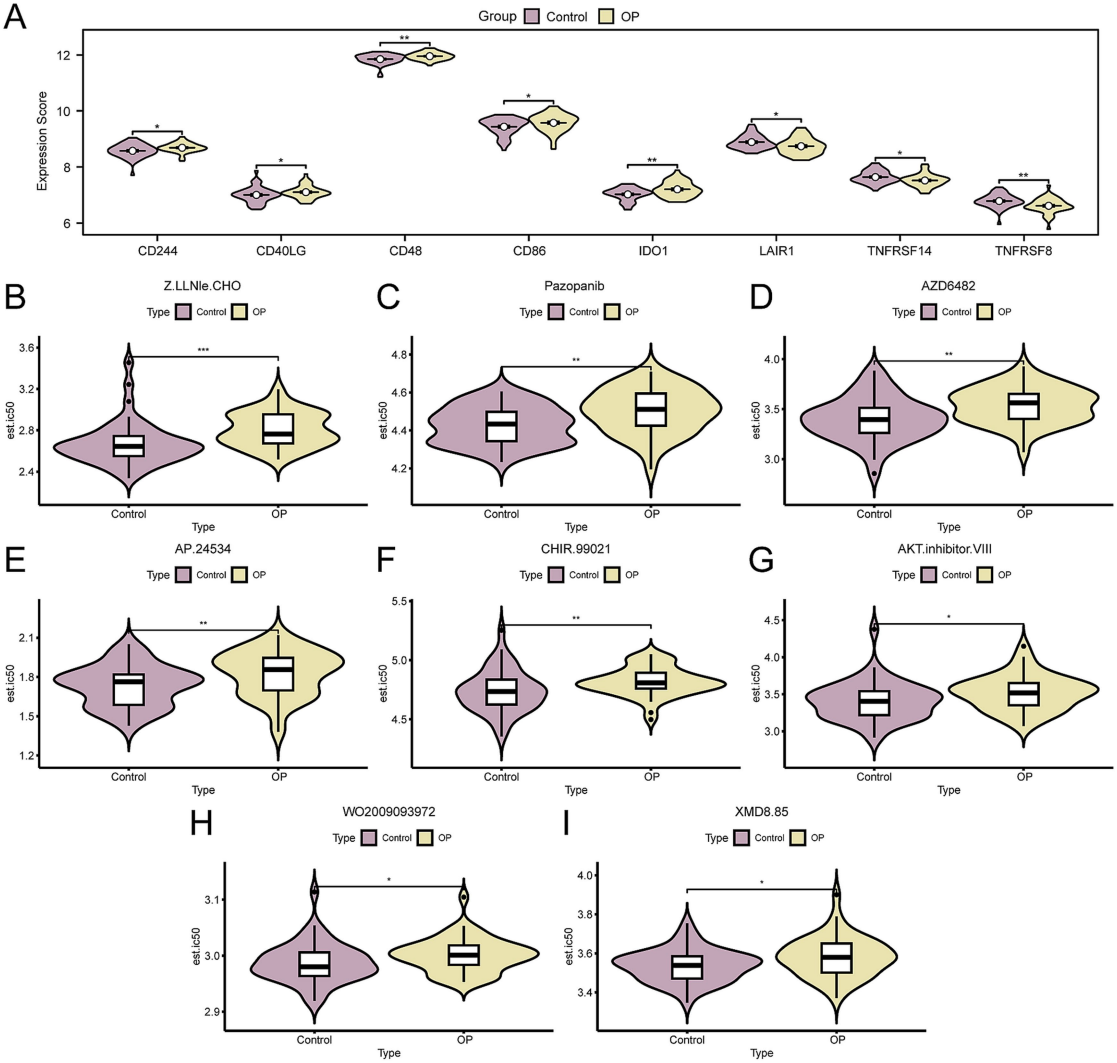
Immune checkpoint genes and drug sensitivity analysis. **(A)** Group comparison of ICGs in OP and control group of the CDs. B–I. Group comparison plot of sensitivity analysis results of OP and control group to drugs Z.LLNle.CHO **(B)**, pazopanib **(C)**, AZD6482 **(D)**, AP.24534 **(E)**, CHIR.99021 **(F)**, AKT.inhibitor.VIII **(G)**, WO2009093972 **(H)**, and XMD8.85 **(I)** based on the GDSC database. OP, osteoporosis; GDSC, genomics of drug sensitivity in cancer; ICGs, immune checkpoint genes; CDs, combined datasets. * *p*- value < 0.05, which is statistically significant; ** *p*- value < 0.01, highly statistically significant; *** *p*-value < 0.001, highly statistically significant. Purple represents the control and yellow represents the OP groups.

We extracted ICGs from the published literature. Upon intersecting these with the genes present in the CDs, we identified a matrix comprising 26 ICGs along with their respective expression levels, as delineated in Supplementary Table S8. Following that, we utilized the Mann–Whitney U test to determine the statistical variance in the expression levels of ICGs between OP and control groups, as depicted in Figure 6A. The analysis identified eight ICGs—*CD48*, *IDO1*, *TNFRSF8*, *CD244*, *CD40LG*, *CD86*, *LAIR1*, and *TNFRSF14*—with significant differences in expression (*p*-value < 0.05) between the groups.

Subsequently, to explore therapeutic strategies for OP patients following mRNA vaccination, we employed the GDSC database’s drug sensitivity information to predict the susceptibility of the OP and control group in the CDs to various drugs. Subsequently, we applied the Mann–Whitney U test (also known as the Wilcoxon rank sum test) to evaluate disparities in drug sensitivity, with the results visualized using grouped comparison plots. The results indicated that eight drugs, including Z.LLNle.CHO, Pazopanib, AZD6482, AP.24534, CHIR.99021, AKT.inhibitor.VIII, WO2009093972, and XMD8.85, showed significant differences in sensitivity between OP and the control group (p-value < 0.05). Moreover, it was observed that the sensitivity to these eight drugs was generally lower in the OP group compared to the control group (Figures 6B–I). Based on the aforementioned findings, it is inferred that OP patients might require higher doses of medication to achieve therapeutic effects, underscoring the significance of personalized treatment strategies in drug dosage selection to ensure efficacy and minimize unnecessary side effects.

### Immune infiltration analysis of OP

**Figure 7:**
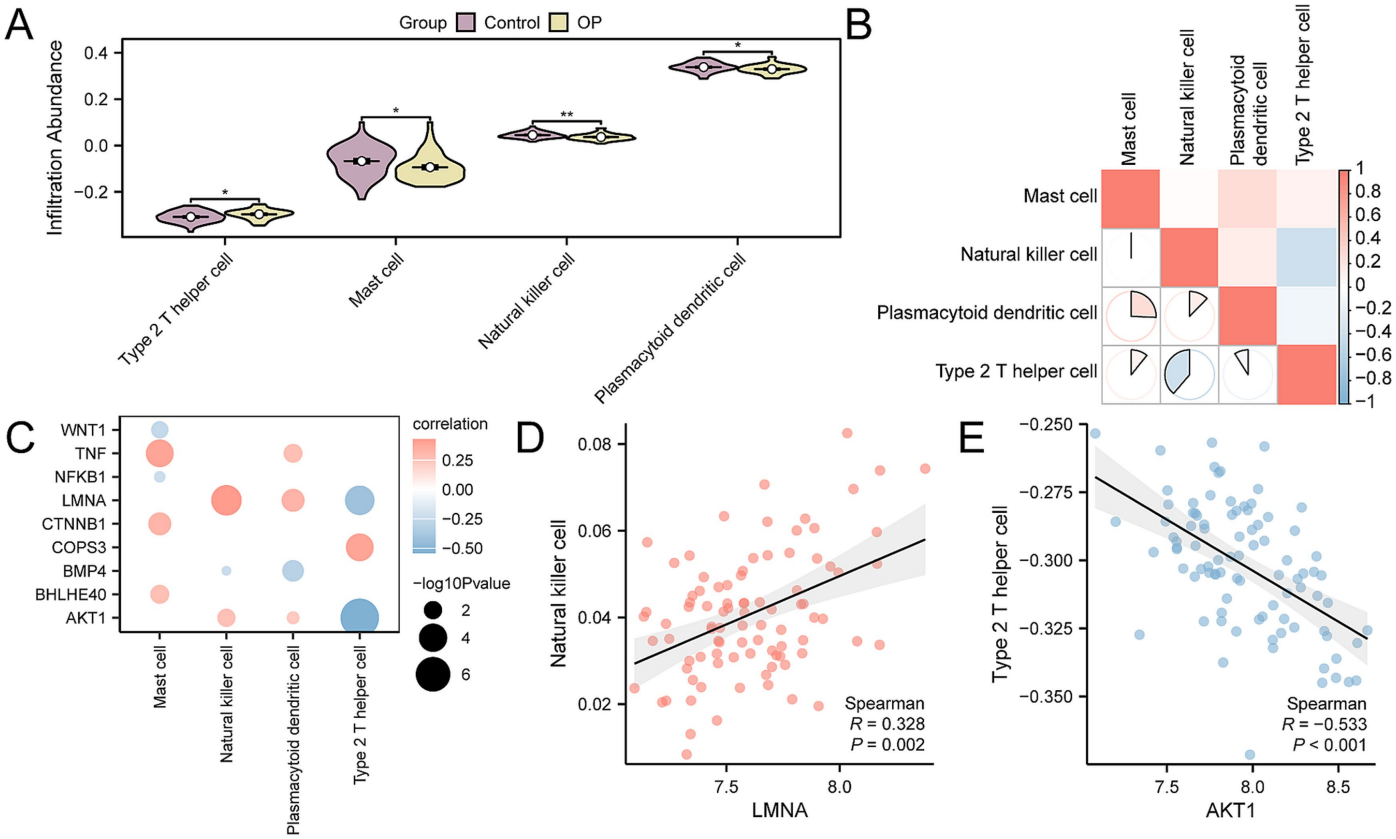
CDs immune infiltration analysis by ssGSEA algorithm. **(A)** Group comparison of immune cells between OP and control group in the CDs. **(B)** Correlation heatmap of immune cell infiltration abundance in the CDs. **(C)** Dot plot of correlation between key genes and immune cell infiltration abundance in the CDs. **(D)** Scatter plot of correlation between top 1 positively related key genes and immune cells. **(E)** Scatter plot of correlation between top 1 negatively related key genes and immune cells. ssGSEA, single-sample gene-set enrichment analysis; OP, osteoporosis; CDs, combined datasets. * *p*-value < 0.05, statistically significant; ** *p*-value < 0.01, highly statistically significant. The absolute value of the correlation coefficient (*r*-value) is weak or no correlation below 0.3, weak correlation between 0.3 and 0.5, moderate correlation between 0.5 and 0.8, and strong correlation above 0.8. In the group comparison chart, purple is the control group, and yellow is the OP group. The correlation heatmap is red for positive correlation and blue for negative correlation.

Using ssGSEA, we quantified the levels of immune cell infiltration for 28 distinct immune cell types in the CDs, comparing the OP group with the control group patients. The findings revealed that the levels of infiltration for natural killer cells, type 2 T helper cells, mast cells, and plasmacytoid dendritic cells were significantly different between the two groups, with a *p*-value < 0.05 (Figure 7A). We performed an analysis to examine the correlation among the four types of immune cells. The results showed that type 2 T helper and natural killer cells had the strongest negative correlation (r-value = −0.387) (Figure 7B). Finally, we selected key genes that were significantly correlated with immune cells (p-value < 0.05). We displayed the overall correlation results through a correlation dot plot (Figure 7C) and the top 1 positively correlated and top 1 negatively correlated key genes were emphasized (Figures 7D,E). The findings indicated that the *LMNA* showed the strongest positive correlation with natural killer cells (r-value = 0.328), whereas the gene *AKT1* had the strongest negative correlation with type 2 T helper cells (r-value = −0.533).

### Establishment of a diagnostic model of OP

**Figure 8:**
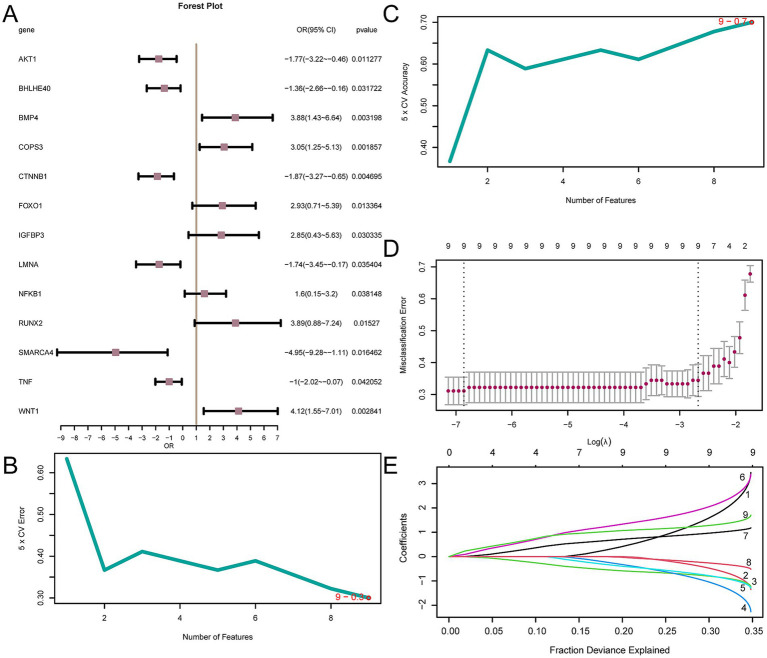
Diagnostic model of OP. **(A)** Forest plot of 13 AP&OG&AGRDEGs included in the logistic regression model in OP diagnostic model. **(B,C)** The number of genes with the lowest error rate **(B)** and the highest accuracy **(C)** obtained by the SVM algorithm are visualized. **(D,E)** Diagnostic model plot **(D)** and variable trajectory plot **(E)** of the LASSO regression model. LASSO (Least Absolute Shrinkage and Selection Operator) regression is a variable selection method that introduces L1 regularization to shrink some coefficients to zero, thereby selecting the most relevant features. OP, osteoporosis; AP&OG&AGRDEGs, Autophagy&Osteogenic&Adipogenic-Related differentially expressed genes; SVM, support vector machine; IncNodePurity, increase in node purity; LASSO, least absolute shrinkage and selection operator.

To determine the diagnostic potential of the 14 AP&OG&AGRDEGs for OP, we initially developed a logistic regression model, which was then depicted through a forest plot (Figure 8A). The analysis showed that 13 of these AP&OG&AGRDEGs were significantly associated with the OP in the logistic regression model, all having *p*-values < 0.05. Subsequently, an SVM model was constructed utilizing the 13 AP&OG&AGRDEGs, which resulted in the minimal error rate as depicted in Figure 8B, and the maximal accuracy as shown in Figure 8C, based on the count of genes. It was shown that when the gene count was nine (AP&OG&AGRDEGs being *NFKB1*, *BHLHE40*, *CTNNB1*, *AKT1*, *LMNA*, *WNT1*, *BMP4*, *TNF*, and *COPS3*), the SVM model attained the highest level of accuracy. Subsequently, the nine genes were used to construct a LASSO regression model, which serves as a diagnostic model for OP. The visualization process involved the depiction of the LASSO regression model (Figure 8D) along with the LASSO coefficient path diagram (Figure 8E). The nine AP&OG&AGRDEGs selected by the LASSO regression model are considered pivotal for diagnosis.

### Validation of OP diagnostic model

**Figure 9:**
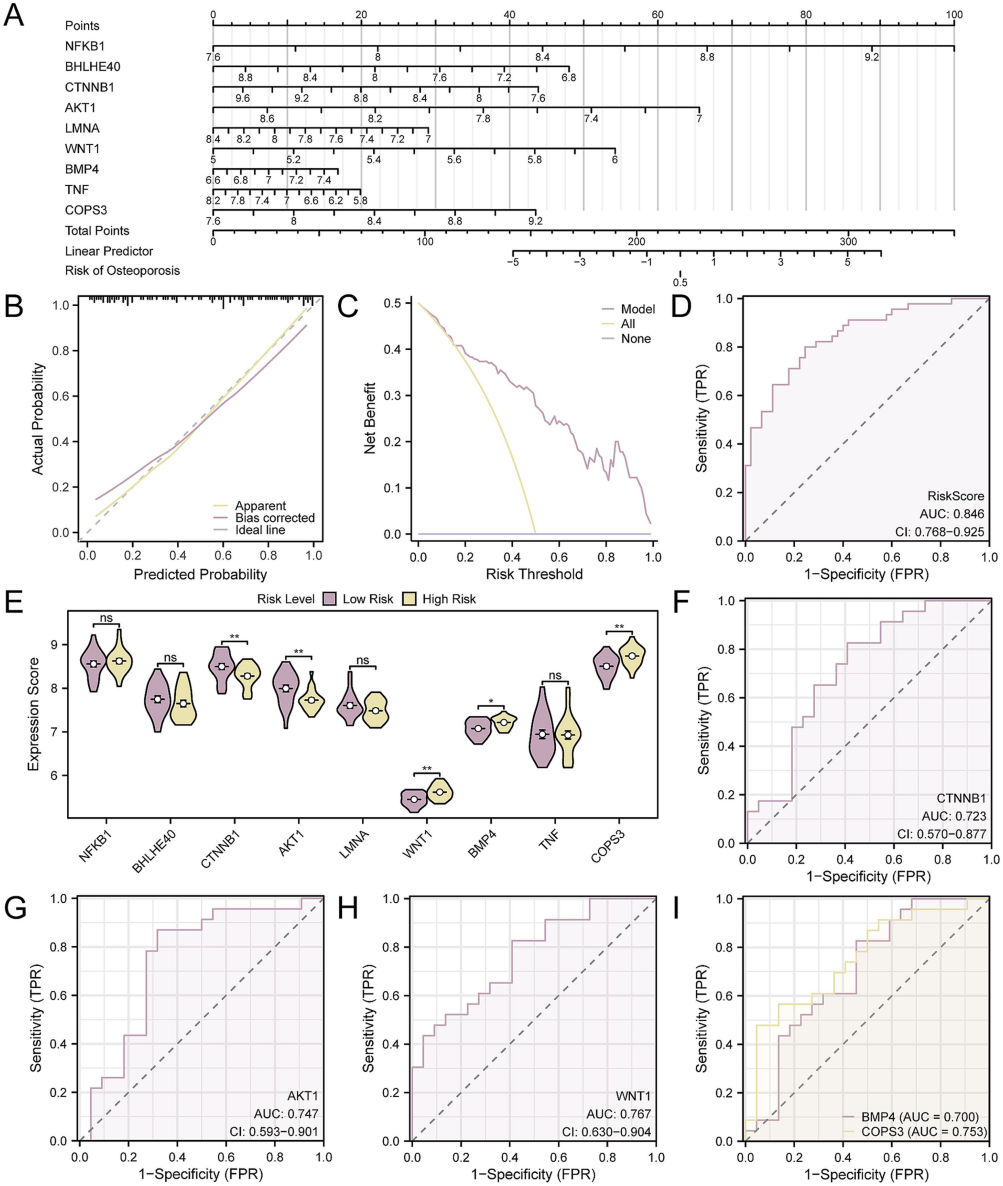
Diagnostic and validation analysis of OP. **(A)** Nomogram of nine key genes in the OP diagnostic model. **(B,C)** Calibration curve **(B)** and DCA plot **(C)** of the OP diagnostic model based on key genes. **(D)** ROC curve of RiskScore in CDs. **(E)** Group comparison of key genes in high- and low-risk groups of OP samples. **(F–I)**. ROC curves of key genes *CTNNB1* **(F)**, *AKT1* **(G)**, *WNT1* **(H)**, *BMP4,* and *COPS3* **(I)** whose expression values were significantly different between high- and low-risk groups of OP samples. The vertical axis of the calibration curve plot represents net benefit, while the horizontal axis represents threshold probabilities. OP, osteoporosis; DCA, decision curve analysis; AUC, the area under the curve. Ns represents p-value ≥ 0.05, not statistically significant; * *p*-value < 0.05, statistically significant; ** *p*-value < 0.01, highly statistically significant. The AUC was accurate between 0.7 and 0.9. In the group comparison chart, purple represents the low-risk and yellow represents the high-risk groups.

To provide additional validation for the OP diagnostic model’s utility, a nomogram was utilized to illustrate the relationships among the key genes across the CDs (Figure 9A). The analysis demonstrated that the expression of *NFKB1* had a significantly greater predictive value for the OP diagnostic model when compared to other genes. Conversely, *BMP4’s* expression level was found to have a substantially lower predictive value relative to the other genes. Next, the diagnostic model’s calibration curve indicated that while the purple calibration line slightly deviated from the perfect model curve (the diagonal), it was still roughly aligned (Figure 9B). Additionally, the DCA outcomes revealed that the model’s simulated curve was consistently above the all-positive and all-negative threshold lines within a specific range, signifying a higher net benefit and thus the model’s enhanced effectiveness (Figure 9C). Moreover, the ROC curve (Figure 9D) indicated that the RiskScore, based on the expression levels of key genes, provides a significant measure of diagnostic accuracy for OP, with an AUC indicating a moderate to high level of accuracy (0.7 < AUC < 0.9). The formula for calculating the RiskScore is as follows:


$$\begin{array}{l} \mathrm{risk}\mathrm{Score}=\mathrm{NFKB}1*\left(0.560\right)+\mathrm{BHLHE}40*\left(-0.029\right)+\\ \mathrm{CTNNB}1*\left(-0.616\right)+AKT1*\left(-0.421\right)+\\ \mathrm{LMNA}*\left(-0.450\right)+WNT1*\left(1.422\right)+\\ BMP4*\left(0.756\right)+TNF*\left(-0.070\right)+\\ \mathrm{COPS}3*\left(1.100\right) \end{array}$$

Using the median RiskScore as a cut-off, the OP samples were divided into high- and low-risk groups. In our analysis of OP samples, we employed box plot comparisons to delineate the expression levels of nine key genes, distinguishing between the high- and low-risk groups. The findings, depicted in Figure 9E, indicated that there were statistically significant differences in expression levels for five key genes when comparing the groups, with a *p*-value < 0.05, including *CTNNB1*, *AKT1*, *WNT1, COPS3*, and *BMP4*. Finally, the ROC curves (Figures 9F–I) confirmed that the expressions of the five key genes in OP samples achieved a considerable degree of accuracy in classifying the groups, as evidenced by an AUC that signifies a notable level of diagnostic effectiveness (0.7 < AUC < 0.9).

### Analysis of immune infiltration in high- and low-risk groups

**Figure 10:**
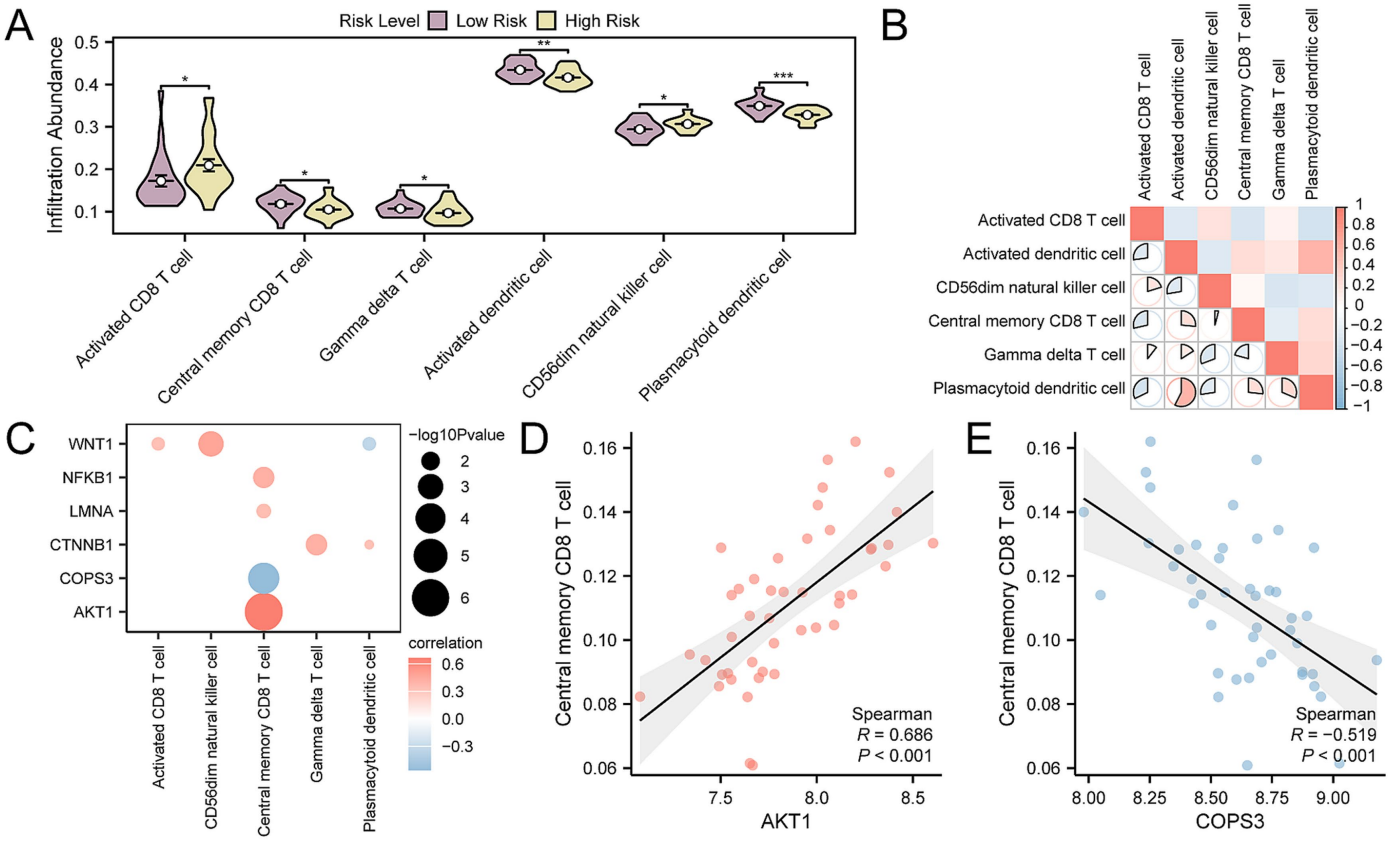
Risk group immune infiltration analysis by ssGSEA algorithm. **(A)** Group comparison of immune cells in OP disease subtypes. **(B)** Heatmap of the correlation of immune cell infiltration abundance in OP samples. **(C)** Dot plot of correlation between key genes and immune cell infiltration abundance in OP samples. **(D)** Scatter plot of correlation between top 1 positively related key genes and immune cells. **(E)** Scatter plot of correlation between top 1 negatively related key gene and immune cells. ssGSEA, single-sample Gene-Set Enrichment Analysis; OP, osteoporosis. * *p*-value < 0.05, statistically significant; ** *p*-value < 0.01, highly statistically significant; *** *p*-value < 0.001, highly statistically significant. The absolute value of the correlation coefficient (r-value) is weak or no correlation below 0.3, weak correlation between 0.3 and 0.5, moderate correlation between 0.5 and 0.8, and strong correlation above 0.8. In the group comparison chart, purple is the low- and yellow is the high-risk group. The correlation heat map is red for positive correlation and blue for negative correlation.

By applying ssGSEA, we evaluated the abundance of immune infiltration across 28 specific immune cell types in OP patients, divided into high- and low-risk categories. The findings indicated that there were statistically significant differences in the abundance of six immune cell types between the groups, with a p-value < 0.05. These cell types were identified as plasmacytoid dendritic cells, activated dendritic cells, activated CD8 + T cells, central memory CD8 + T cells, gamma delta T cells, and CD56dim natural killer cells (Figure 10A), In addition, the correlation analysis of the infiltration levels among the six immune cell types in OP samples highlighted a significant positive correlation, particularly between plasmacytoid dendritic cells and activated dendritic cells, with an r-value = 0.574 (Figure 10B). Finally, we screened for key genes that showed significant correlation with immune cells (*p*-value < 0.05) and presented the overall correlation results (Figure 10C) and the top 1 positively correlated and top 1 negatively correlated key genes through correlation dot plots (Figures 10D,E). The results indicated that *AKT1* exhibited the strongest positive correlation with central memory CD8 T cell (r-value = 0.686), while *COPS3* exhibited the most pronounced negative association with central memory CD8 T cell (r-value = −0.519).

### Verification and correlation analysis of key gene expression differences

**Figure 11:**
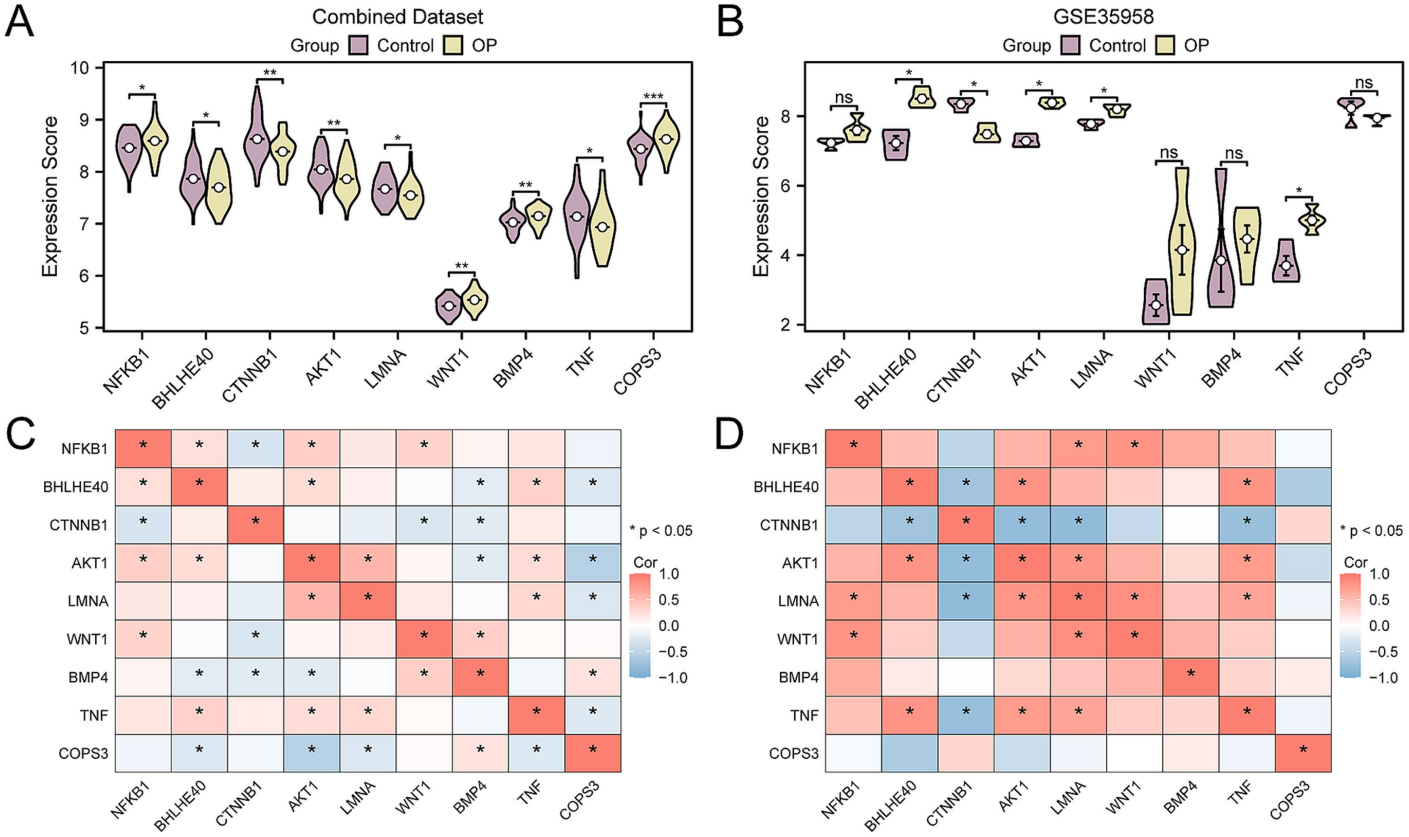
Correlation and expression difference analysis for key genes. **(A)** Group comparison of key genes in the CDs. **(B)** Group comparison of key genes in dataset GSE35958. **(C)** Correlation heatmap of key genes in the CDs. **(D)** Correlation heatmap of key genes in GSE35958. OP, osteoporosis; CDs, combined datasets. Ns represents *p*-value ≥ 0.05, not statistically significant; * *p*-value < 0.05, statistically significant; ** *p*-value < 0.01, highly statistically significant; *** *p*-value < 0.001, highly statistically significant. The absolute value of the correlation coefficient (r-value) is weak or no correlation below 0.3, weak correlation between 0.3 and 0.5, moderate correlation between 0.5 and 0.8, and strong correlation above 0.8. In the group comparison chart, purple is the control, and yellow is the OP group. The correlation heatmap shows a positive correlation in red and a negative correlation in blue.

To further authenticate the differential expression of key genes among groups within the CDs and the validation dataset GSE35958, we presented comparative expression graphs. The findings indicated that there was a statistically significant difference in the expression levels of nine key genes between the OP and control group within the CDs, with a *p*-value < 0.05 (Figure 11A). Simultaneously, the expression levels of five key genes were statistically significant in the validation dataset GSE35958 (*p*-value < 0.05) (Figure 11B), including *BHLHE40*, *CTNNB1*, *AKT1*, *LMNA*, and *TNF*. Subsequently, the correlation between the expression levels of key genes was determined for the combined data sets and GSE35958, applying the Spearman correlation technique for the analysis. The results showed that *AKT1* and *BHLHE40*, *TNF* and *BHLHE40*, *LMNA* and *AKT1*, *TNF* and *AKT1*, *TNF* and *LMNA* all exhibited positive correlation in both the combined and the validation datasets GSE35958 (Figures 11C,D).

### Construction of regulatory networks

**Figure 12:**
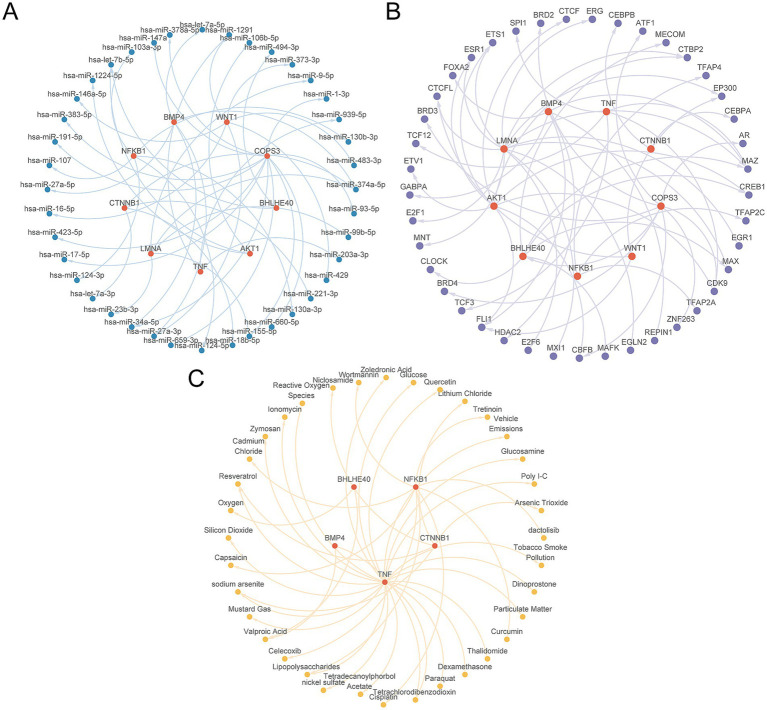
Regulatory network of key genes. **(A)** The mRNA-miRNA regulatory network of key genes. **(B)** mRNA-TF regulatory network of key genes. **(C)** mRNA-Drug regulatory network of key genes. TF, transcription factor. Red is mRNA, blue is miRNA, purple is TF, and yellow is Drug.

First, we obtained 40 miRNAs related to the nine key genes through the TarBase database, with specific information provided in Supplementary Table S9. Next, we retrieved 42 TFs associated with the nine key genes from the ChIPBase database, with detailed information available in Supplementary Table S10. Additionally, employing the CTD database, we ascertained 37 candidate drugs or molecular compounds linked to five key genes, with specific information listed in Supplementary Table S11. To culminate the analysis, we used Cytoscape software (V3.7.0) to generate and illustrate the interaction networks among mRNA, miRNA, TFs, and drugs (Figures 12A–C).

### Construction of subtypes of OP

**Figure 13:**
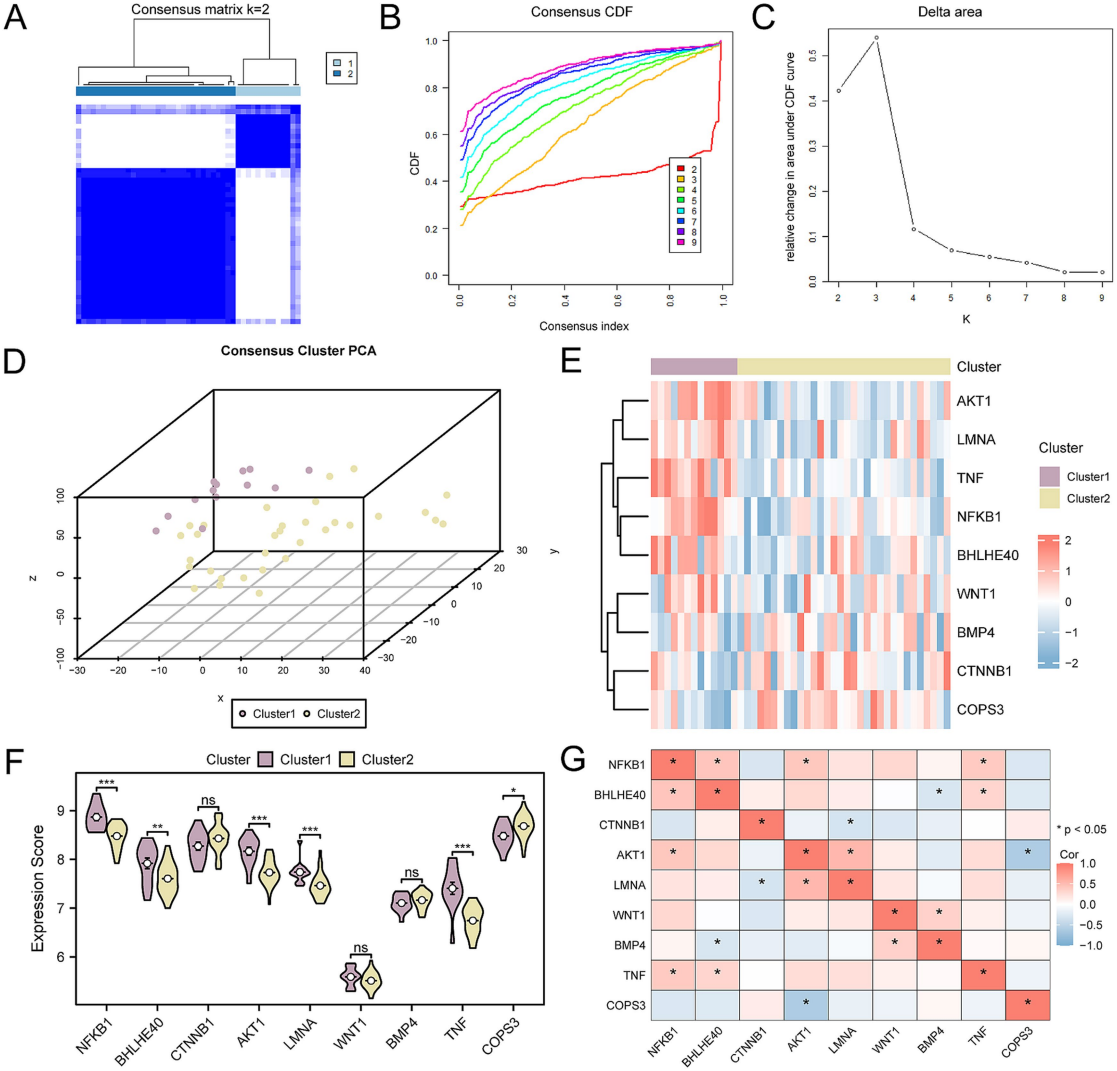
Consensus clustering analysis for key genes. **(A)** Concordance cluster results plot for OP samples. **(B,C)** Concordance cumulative distribution function (CDF) plot **(B)** and Delta plot **(C)** for concordance cluster analysis. **(D)** 3D PCA plot of two subtypes of OP. **(E)** Heatmap of the association of key genes. **(F)** Group comparison chart of key genes. **(G)** Heatmap of the association of key genes in OP samples. OP, osteoporosis; CDF, Empirical Cumulative Distribution Function; PCA, Principal Component Analysis. ns means p-value ≥ 0.05, not statistically significant; * *p*-value < 0.05, statistically significant; *** *p*-value < 0.001, highly statistically significant. The absolute value of the correlation coefficient (*r*-value) is weak or no correlation below 0.3, weak correlation between 0.3 and 0.5, moderate correlation between 0.5 and 0.8, and strong correlation above 0.8. Purple is subtype A (Cluster 1) and yellow is subtype B (Cluster 2). The correlation heatmap shows positive (in red) and negative (in blue) correlations.

In probing the OP disease subtypes present in the CDs, we utilized the R package ConsensusClusterPlus. It harnessed the expression profiles of the nine key genes within OP samples to identify distinct OP-related disease subtypes through a consensus clustering approach. Ultimately, two subtypes were determined: Subtype A (Cluster 1) and Subtype B (Cluster 2) (Figures 13A–C). Among them, Subtype A includes 13 samples, and Subtype B includes 32 samples. The 3D PCA plot highlighted significant differences between the two disease subtypes (Figure 13D). Next, leveraging the R package pheatmap, we produced a heatmap to visually represent a differential expression of key genes among the two OP subtype categories (Figure 13E). Continuing our investigation, we engaged in an assessment to identify the differences in the expression level of key gene between the two OP subtypes. The results indicated that six out of the nine key genes exhibited statistically significant differences in expression between the two subtypes (*p*-value < 0.05). These genes included: *NFKB1, AKT1, LMNA, TNF, BHLHE40*, and *COPS3* (Figure 13F). Finally, the study on the correlation of key genes within osteoporotic samples indicated that *NFKB1* correlates positively with *BHLHE40*, *AKT1* with *NFKB1*, *TNF* with *NFKB1*, *TNF* with *BHLHE40*, *LMNA* with *AKT1*, and BMP4 with WNT1. Negative correlations were identified between *BMP4* and *BHLHE40*, *LMNA* and *CTNNB1*, and *COPS3* with *AKT1* (Figure 13G).

### Analysis of immune infiltration in subtypes of OP

**Figure 14:**
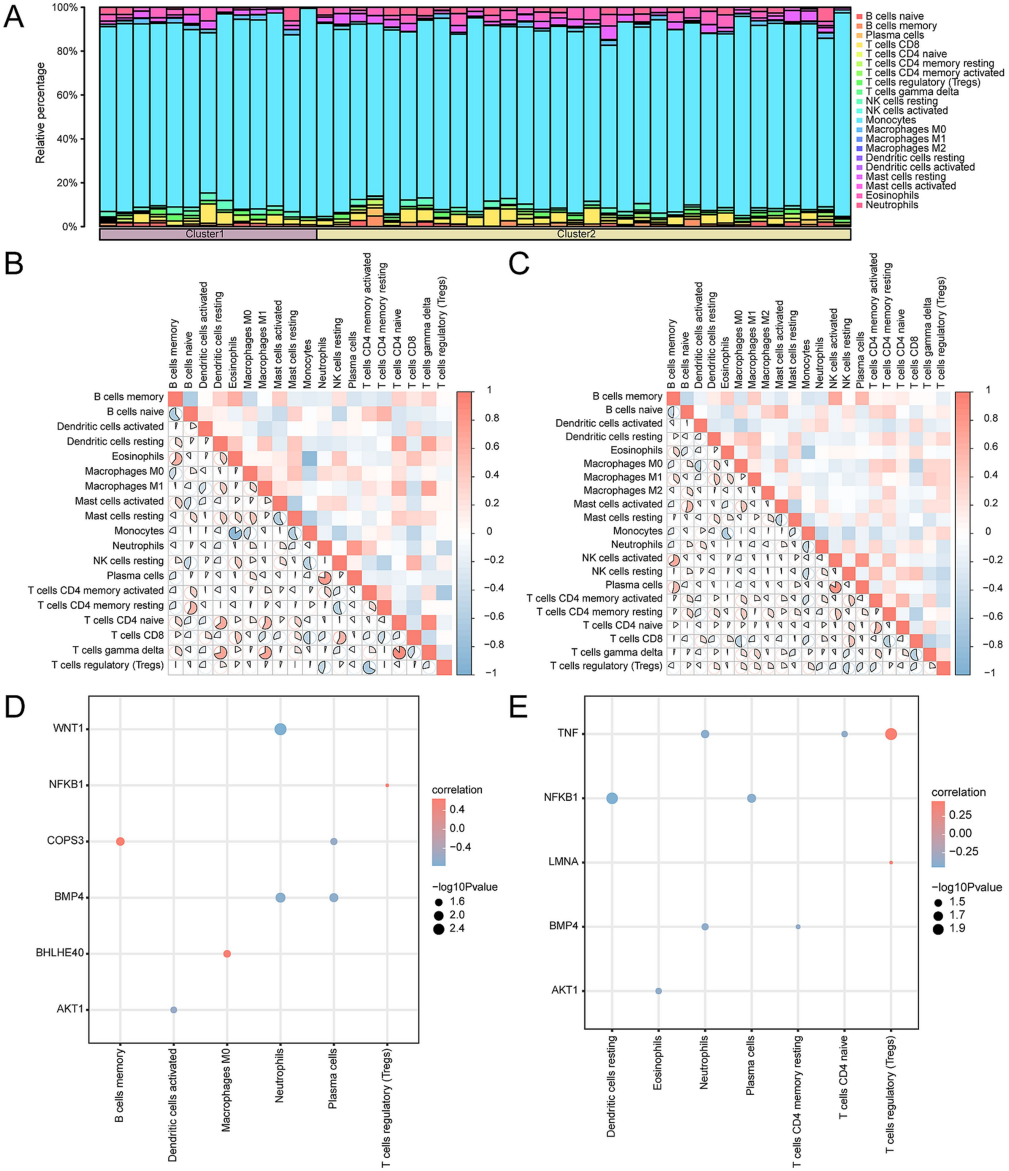
Consensus cluster immune infiltration analysis by CIBERSORT algorithm. **(A)** Histogram of immune cell infiltration abundance in OP subtypes. **(B,C)** Heatmap of correlation of immune cell infiltration abundance in subtype A **(B)** and subtype B **(C)**. **(D,E)** Dot plots of correlation of key genes with immune in filtration abundance in subtype A **(D)** and subtype B **(E)**. OP, osteoporosis. The absolute value of the correlation coefficient (*r*-value) is weak or no correlation below 0.3, weak correlation between 0.3 and 0.5, moderate correlation between 0.5 and 0.8, and strong correlation above 0.8. The component bargraph is purple for subtype A and yellow for subtype B. The correlation heatmap is red for positive and blue for negative correlation.

Employing the CIBERSORT algorithm, we assessed the relationships between 22 categories of immune cells and the two OP subtypes: Subtype A (Cluster 1) and Subtype B (Cluster 2). Following the analysis of immune cell infiltration, we generated a bar chart illustrating the relative frequencies of these immune cells within the samples of the CDs (Figure 14A). Further along in our study, we explored the correlation of immune cell infiltration abundance between samples classified as OP Subtype A and as Subtype B (Figures 14B,C). This analysis identified strong positive correlations in Subtype A, specifically between T cells gamma delta and T cells CD4 naïve (*r*-value = 0.884), between neutrophils and plasma cells (*r*-value = 0.786), and between eosinophils and B cells memory (*r*-value = 0.609). Monocytes and eosinophils (*r*-value = −0.85), B cells naïve and B cells memory (*r*-value = −0.603), and monocytes and mast cells resting (*r*-value = −0.564) showed negative correlations. Within Subtype B, significant positive associations were found between plasma cells and activated NK cells (*r*-value = 0.836), activated NK cells and memory B cells (*r*-value = 0.648), and plasma cells and memory B cells (*r*-value = 0.540). Conversely, negative correlations were noted between resting CD4 Memory T cells and CD8 T cells (*r*-value = −0.527), and CD8 T cells and macrophages M0 (*r*-value = −0.510). In the final phase of our study, we investigated the correlation between the expression of key genes and the abundance of immune cell infiltration in OP Subtype A and Subtype B (Figures 14D,E). For Subtype A, the strongest positive association was found between the gene *COPS3* and memory B cells (*r*-value = 0.647). The most significant inverse relationship was between *WNT1* and neutrophils (*r*-value = −0.770). In Subtype B, the gene *TNF* had the most substantial positive correlation with T regulatory cells (Tregs) (r-value = 0.456); the gene *NFKB1* had the most pronounced negative correlation with resting dendritic cells (*r*-value = −0.450).

## Discussion

OP continues to be a major health issue that affects patients physically and emotionally and carries high care costs (47). Because the symptoms are not noticeable at the beginning, OP is often only diagnosed after an osteoporotic fracture occurs; however, by this stage, the therapeutic effectiveness may have significantly declined. Even with advances in anti-OP treatments, including targeted molecular therapies, discontinuation of denosumab results in rapid BMD loss, and an increased risk of hypercalcemia and multiple vertebral fractures according to recent data (48, 49). The use of romosozumab is only approved in some countries, and following the cardiovascular concerns seen in romosozumab clinical trials, the safety reports for the drug, especially cardiovascular events, were of concern (50). Therefore, in this study, we established a gene network associated with OP to deepen our understanding of its immune response and provide new insights for diagnosis and treatment. We also highlighted the potential of personalized therapy and immunomodulation in the treatment of OP.

It is well known that inflammatory response is closely related to the occurrence and development of OP. The GSEA results show that DEGs are predominantly enriched in inflammation-related pathways, such as the IL-6 signaling pathway, and similar. There is substantial *in vitro* and *in vivo* experimental evidence that confirms this result (51). In addition, the immune status correlates closely with the inflammatory response (52). Subsequent ICGs analysis revealed statistically significant differential expression of eight genes in OP patients. *IDO1,* an ICG, is activated in a state of chronic inflammation; studies suggest that it triggers inhibitory signal transduction by binding to PI3K p110 and SHP-1, promoting immunosenescence, impairing autophagy, and contributing to the development of OP (53). *CD40LG*, or *CD40L*, belongs to the tumor necrosis factor receptor superfamily member 5 and induces B cells to secrete immunoglobulins (54). An *in vivo* experiment showed that the silencing of *CD40L* attenuated the ability of OVX (ovariectomy) to stimulate OC-mediated bone resorption and induce bone loss. In other words, *CD40L* promotes bone resorption by stimulating the formation of OC (55); this result is predicated on the premise of T-cell depletion.

The expression of key genes might have some associations with immune cells. This research showed that the *LMNA* has the strongest positive correlation with natural killer (NK) cell (Figure 7D), it suggested that nuclear envelope structural proteins may influence NK cell function through epigenetic regulation. As a major component of the nuclear lamina, upregulated *LMNA* expression could alter the chromatin spatial conformation of NK cells. NK cells are notable for their expression of macrophage colony-stimulating factor (*M-CSF*) and receptor activator of nuclear factor-kappa B ligand (*RANKL*), with *RANKL* being instrumental in the activation and differentiation of OC, highlighting a role for NK cells in osteolytic processes (56), suggesting that NK cells contribute to the process of bone erosion. Furthermore, the significant negative correlation between NK cells and Th2 cells (7B) indicated a competitive regulatory interplay among immune subsets in the OP microenvironment. The activation process of Th2 cells involves the release of various cytokines, including IL-4. IL-4 has been confirmed by *in vitro* and in vivo experiments to promote bone regeneration and prevent bone loss (57). Th2 cells may antagonize the pro-osteoclastic effects of NK cells via anti-inflammatory cytokines like IL-4. The absence of *AKT1* enhances osteoblast differentiation and negatively regulates osteoblast differentiation. However, *AKT1* promotes the differentiation of OC (58). In our study, *AKT1* has the strongest negative correlation with Th2 cells (Figure 7C), which implies indirect regulation of the osteoblast–osteoclast balance via AKT1 signaling. AKT1 inhibitors, such as MK-2206 (in clinical trials for cancer), have been shown to play a significant role in suppressing osteoclast differentiation (59). Therefore, *AKT1* inhibitors might be potential drugs for treating OP. This finding is important as it guides future in-depth research.

We conducted GO and KEGG enrichment analyses on a total of 14 AP&OG&AGRDEGs. The outcomes from the BP component of the GO analysis revealed that the “regulation of cell death induced by oxidative stress” is implicated in the onset and progression of OP. By suppressing oxidative stress, *FOXO1* inhibits osteoblast apoptosis (60). However, the function of *FOXO1* is still controversial. It is widely recognized that Runt-related transcription factor 2 (*Runx2*) is a crucial transcription factor for osteoblast differentiation and the process of bone formation; a deficiency in *Runx2* could result in a complete failure of bone formation. Through the inhibition of *Runx2* in osteoblasts, *FOXO1* regulates the expression of osteocalcin in a manner that is contingent upon the insulin-like growth factor 1 (*IGF1*) and insulin signaling pathways (61). In other words, *FOXO1* is a negative regulator of the osteoblast-specific transcription factor *Runx2*.

Subsequently, we constructed diagnostic models for OP. As a result, nine key diagnostic genes were identified, including *AKT1, NFKB1, TNF, CTNNB1, LMNA, BHLHE40, BMP4, WNT1, and COPS3.* Through nomogram analysis, it was found that the expression level of *NFKB1* has the highest utility for the OP diagnostic model. Bortezomib, a proteasome inhibitor of the *NFKB1* - regulated inflammatory pathway, is widely used to treat multiple myeloma. It has been reported to directly stimulate osteoblast growth and differentiation while inhibiting osteoclast development and activity, thereby exerting osteogenic effects in clinical settings (62). The RiskScore analysis revealed a markedly significant statistical difference in the infiltration levels of plasmacytoid dendritic cells between the high- and low-risk groups within the OP samples (Figure 10A). Dendritic cells (DCs) are integral to the inflammatory processes that lead to osteoclastogenesis and are linked to the development of inflammatory bone diseases. In the absence of estrogen, DCs have a longer lifespan and express higher levels of *IL-7* and *IL-15*, together inducing memory T cells to produce IL-17A and TNF-*α*. The resulting cytokines drive inflammation-mediated bone loss (63). It appears, consistent with our results, that memory T cells are also involved in the process of OP. In the final validation model, *BHLHE40*, *CTNNB1*, *AKT1*, *LMNA*, and *TNF* were found to be differentially expressed in the validation dataset. In other words, their likelihood of serving as diagnostic markers is higher. Literature suggests that elevated *BHLHE40* expression is tightly connected with osteoclast maturation. *In vitro* experiments confirm that *BHLHE40* stimulates osteoclast development, and in contrast, its absence *in vivo* is linked to greater bone density and lessened osteoclast formation (64). Although some results have been confirmed, our blood - based mRNA diagnostic model, though adaptable for clinical screening via RT - qPCR or NanoString, needs further validation across diverse ethnicities and age groups and integration with BMD and FRAX scores to optimize risk stratification before it can be generalized.

The TFs could promote or inhibit the transcription process of mRNA by recognizing and binding to specific DNA sequences. Further, miRNAs exert regulatory control over gene expression by binding to the 3′ untranslated region (3’ UTR) of their target mRNAs, resulting in mRNA degradation or the inhibition of their translation into proteins. Thus, TFs and miRNA play a major role in regulating gene expression. Within the scope of this study, we identified 40 miRNAs that correspond to the nine key genes. The *miR-483-3p* is one of the miRNAs we have predicted. Previous studies have indicated a correlation between *miR-483-3p* and OP associated with estrogen deficiency (65). Zhou et al. found that elevated levels of *miR-483-3p* significantly boosted the expression of mRNA transcripts related to osteogenic markers, specifically alkaline phosphatase (*ALP*), *Runx2*, and osteocalcin (*OCN*). Additionally, the upregulation of *miR-483-3p* significantly elevated the expression levels of Wnt1 and *β*-catenin. As a result, the study suggested that *miR-483-3p* could potentially augment osteoblast proliferation and osteogenesis by activating the Wnt/β-catenin signaling pathway (66). These findings are in alignment with our predictive outcomes, implying the credibility of our predictive results.

As demonstrated by the drug sensitivity results in our study, it is also important to emphasize personalized treatment strategies for OP patients to ensure therapeutic effectiveness and minimize unnecessary side effects. We have conducted a further subtype analysis of the disease among OP patients. Ultimately, we have delineated two distinct disease subtypes, characterized by significant differential expression of six pivotal genes across these subtypes: *NFKB1*, *AKT1*, *LMNA*, *TNF*, *BHLHE40*, and *COPS3*. The BHLHE40, also known as human differentiated embryonic chondrocyte expressed gene 1 (*DEC1*), is believed to be related to enhanced osteogenesis. After the knockout of *DEC1, DEC1*-deficient mice exhibited at four and 24 weeks of age a phenotype of reduced bone mass compared to age-matched wild-type (WT) mice. Moreover, the bone mass decay was more pronounced in the 24-week-old mice group (67). However, other researchers have found that *BHLHE40* is associated with osteoclastogenesis and abnormal bone resorption. Mice deficient in *BHLHE40* were resistant to estrogen deficiency-induced OP. In other words, the deficiency of *BHLHE40* increased bone formation. Further mechanistic research elucidated that the bone mass increment in *BHLHE40*-deficient conditions stems from an inherent cellular deficiency affecting osteoclast differentiation in the mice. *BHLHE40* is shown to upregulate the transcription of *FOS* and *NFATC1* genes through direct interaction with their promoter regions. The latter promoted osteoclastogenesis in ovariectomized mice (64). Additionally, drug sensitivity analysis (Figure 6G) revealed reduced AKT inhibitor sensitivity in OP patients, underscoring the need for personalized dosing regimens. Therefore, personalized treatment is necessary. In further subtypes of immune infiltration analysis, TNF and regulatory T cells (Tregs) are positively correlated. *In vitro*, Tregs directly inhibit the differentiation and function of OC (68). In TNF-transgenic mice, a phenomenon of bone loss occurs, which could be reversed by an increase in the number of Tregs (69). In certain types of OP patients, such as postmenopausal OP, the increase in the number of Tregs might serve as a protective immune response to shield the patients from TNF-induced bone loss. This suggests an interaction between key genes and the immune system, possibly affecting the activity and function of immune cells.

However, this study has several limitations. First, although we employed the sva R package for batch effect correction, residual batch effects may still influence gene expression patterns, potentially compromising the reproducibility of certain findings. Second, the relatively small sample size may impact statistical power and the generalizability of the diagnostic model. Despite integrating multiple datasets and conducting rigorous validation, larger cohort studies are required to further confirm the reliability of the results. Finally, this work primarily relies on bioinformatics analyses and lacks experimental validation. Future studies will prioritize validating the expression of key genes using qPCR or Western blot and elucidating their functional roles in OP-related pathways. These additional investigations will enhance the robustness and biological relevance of the findings.

Our diagnostic model offers a novel molecular strategy to refine OP diagnosis by enabling early identification of high-risk populations through peripheral blood gene expression profiling (e.g., qPCR or RNA sequencing) as a complement to BMD testing. This approach holds translational potential for guiding personalized interventions, such as risk stratification systems integrating key genes (*AKT1*, *NFKB1*). However, clinical implementation faces three major challenges. First, biomarkers predicted via bioinformatics require validation in large prospective cohorts, as retrospective public datasets may overestimate diagnostic accuracy due to batch effects and population heterogeneity (e.g., age, comorbidities). Second, the molecular complexity of OP pathogenesis—exemplified by *AKT1*’s pleiotropic roles in bone homeostasis, which are modulated by miRNA-mediated epigenetic regulation or post-translational modifications—cannot be fully resolved by transcriptome-level analysis alone. Third, the model currently lacks specificity for OP subtypes driven by distinct etiologies, such as inflammatory bone loss in rheumatoid arthritis. Addressing these limitations will require integrating multi-omics data (e.g., proteomics, epigenetics) with clinical phenotypes to develop etiology-specific diagnostic algorithms, thereby enhancing both clinical utility and biological relevance.

## Conclusion

This research established a network of genes associated with OP and thoroughly examined the molecular mechanisms of the immune response within the context of OP. The identification of critical diagnostic genes and the analysis of immune cell infiltration have enhanced our profound understanding of OP’s pathophysiology, potentially pointing toward new avenues for refining diagnostic and therapeutic strategies. Furthermore, the study accentuates the significance of tailored therapeutic approaches and highlights the potential role of immunomodulation in the clinical management of OP.

## Data Availability

The original contributions presented in the study are included in the article/Supplementary material, further inquiries can be directed to the corresponding author/s.

## Glossary

OP: Osteoporosis
DEGs: Differentially Expressed Genes
APRGs: Autophagy-Related Genes
OGRGs: Osteogenic-Related Genes
AGRGs: Adipogenic-Related Genes
GSEA: Gene Set Enrichment Analysis
AP&OG&AGRDEGs: Autophagy& Osteogenic&Adipogenic-Related Differentially Expressed Genes
GO: Gene Ontology
KEGG: Kyoto Encyclopedia of Genes and Genomes
SVM: Support Vector Machine
LASSO: Least Absolute Shrinkage and Selection Operator
TF: Transcription Factor
PCA: Principal Component Analysis
CDs: Combined Datasets
GDSC: Genomics of Drug Sensitivity in Cancer
ICGs: Immune Checkpoint Genes
ssGSEA: Single-Sample Gene-Set Enrichment Analysis
DCA: Decision Curve Analysis
AUC: The Area Under the Curve
CDF: Empirical Cumulative Distribution Function
BMD: Bone Mineral Density
IDO1: Indoleamine 2,3-dioxygenase 1
PI3K p110: Phosphatidylinositol-4,5-bisphosphate 3-kinase catalytic subunit alpha isoform
SHP-1: Src homology region 2 domain-containing phosphatase-1
CD40L: CD40 ligand
LMNA: Lamin A/C
AKT1: Protein kinase B alpha
M-CSF: Macrophage colony-stimulating factor
RANKL: Receptor activator of nuclear factor kappa-B ligand
FOXO1: Forkhead box O1
OB: Osteoblast
OC: Osteocalcin
Runx2: Runt-related transcription factor 2
IGF1: Insulin-like growth factor 1
NFKB1: Nuclear factor kappa B subunit 1
BHLHE40: Basic helix–loop–helix family member e40
ALP: Alkaline phosphatase
OCN: Osteocalcin
FOS: FBJ murine osteosarcoma viral oncogene homolog
NFATC1: Nuclear factor of activated T-cells, cytoplasmic 1
