# Supplementary material for: Integrative genomic analysis and diagnostic modeling of osteoporosis: unraveling the interplay of autophagy, osteogenesis, adipogenesis, and immune infiltration
Source: Front Med (Lausanne). 2025 Apr 17;12:1544390. doi: 10.3389/fmed.2025.1544390 (PMC12043663; doi:10.3389/fmed.2025.1544390)
Supplement: Supplementary file 1 [file Data_Sheet_1.ZIP › supplementary tables-s1,s6-7.docx]

**Table S1 GEO Microarray Chip Information**

|  | **GSE62402** | **GSE56815** | **GSE35958** |
| --- | --- | --- | --- |
| Platform | GPL5175 | GPL96 | GPL570 |
| Species | Homo Sapiens | Homo Sapiens | Homo Sapiens |
| Tissue | Blood | Blood | Mesenchymal Stromal Cells |
| Samples in OP group | 5 | 40 | 5 |
| Samples in Normal group | 5 | 40 | 4 |
| Reference | / | PMID：29330445 | PMID：23028809 |

GEO，Gene Expression Omnibus.

**Table S6 Results of GO and KEGG Enrichment Analysis for AP&OG&AGRDEGs**

| ONTOLOGY | ID | GeneRatio | BgRatio | pvalue | p.adjust |
| --- | --- | --- | --- | --- | --- |
| BP | GO:0048660 | 5/14 | 170/18800 | 1.07E-07 | 8.03E-05 |
| BP | GO:0048659 | 5/14 | 173/18800 | 1.17E-07 | 8.03E-05 |
| BP | GO:1901653 | 6/14 | 361/18800 | 1.27E-07 | 8.03E-05 |
| BP | GO:0045913 | 4/14 | 77/18800 | 2.52E-07 | 8.19E-05 |
| BP | GO:1903201 | 4/14 | 77/18800 | 2.52E-07 | 8.19E-05 |
| CC | GO:0005788 | 3/14 | 311/19594 | 0.001266 | 0.039238 |
| CC | GO:0045121 | 3/14 | 326/19594 | 0.00145 | 0.039238 |
| CC | GO:0098857 | 3/14 | 327/19594 | 0.001462 | 0.039238 |
| CC | GO:0005901 | 2/14 | 82/19594 | 0.001524 | 0.039238 |
| CC | GO:0044853 | 2/14 | 113/19594 | 0.002867 | 0.049876 |
| MF | GO:0031994 | 2/14 | 13/18410 | 4.17E-05 | 0.004335 |
| MF | GO:0005520 | 2/14 | 29/18410 | 0.000215 | 0.008548 |
| MF | GO:0043425 | 2/14 | 31/18410 | 0.000247 | 0.008548 |
| MF | GO:0140297 | 4/14 | 470/18410 | 0.000343 | 0.008905 |
| MF | GO:0005125 | 3/14 | 235/18410 | 0.000674 | 0.014011 |
| KEGG | hsa04931 | 5/13 | 108/8164 | 4.37E-07 | 6.81E-05 |
| KEGG | hsa05418 | 5/13 | 139/8164 | 1.54E-06 | 8.88E-05 |
| KEGG | hsa04936 | 5/13 | 142/8164 | 1.71E-06 | 8.88E-05 |
| KEGG | hsa05165 | 6/13 | 331/8164 | 5.72E-06 | 0.000223 |
| KEGG | hsa04211 | 4/13 | 89/8164 | 8.75E-06 | 0.000271 |

GO，Gene Ontology；BP，Biological Process；CC，Cellular Component；MF，Molecular Function；KEGG，Kyoto Encyclopedia of Genes and Genomes；AP&OG&AGRDEGs，Autophagy&Osteogenic&Adipogenic-Related Differentially Expressed Gene

**Table S7 Results of GSEA for Combined Datasets**

| ID | Set Size | Enrichment Score | NES | pvalue | p.adjust | qvalue |
| --- | --- | --- | --- | --- | --- | --- |
| REACTOME_NEUTROPHIL_DEGRANULATION | 263 | -0.46231 | -2.09722 | 2.4E-10 | 4.85E-07 | 4.16E-07 |
| REACTOME_METABOLISM_OF_RNA | 290 | 0.397252 | 1.902469 | 7.1E-09 | 7.16E-06 | 6.14E-06 |
| REACTOME_NGF_STIMULATED_TRANSCRIPTION | 29 | -0.78685 | -2.43097 | 1.15E-08 | 7.73E-06 | 6.63E-06 |
| REACTOME_NUCLEAR_EVENTS_KINASE_AND_TRANSCRIPTION_FACTOR_ACTIVATION | 46 | -0.68224 | -2.30594 | 6.25E-08 | 3.15E-05 | 2.7E-05 |
| REACTOME_SIGNALING_BY_NTRKS | 84 | -0.56582 | -2.17177 | 4.92E-07 | 0.000199 | 0.00017 |
| WP_IL18_SIGNALING_PATHWAY | 187 | -0.4262 | -1.8513 | 9.17E-06 | 0.003084 | 0.002644 |
| WP_HEMATOPOIETIC_STEM_CELL_DIFFERENTIATION | 44 | -0.61748 | -2.0686 | 1.49E-05 | 0.004299 | 0.003686 |
| REACTOME_SIGNALING_BY_RECEPTOR_TYROSINE_KINASES | 333 | -0.35656 | -1.65676 | 2.23E-05 | 0.005613 | 0.004813 |
| REACTOME_TRNA_PROCESSING | 52 | 0.542633 | 2.006914 | 4.4E-05 | 0.008448 | 0.007244 |
| PID_MET_PATHWAY | 47 | -0.5902 | -2.00333 | 4.92E-05 | 0.008448 | 0.007244 |
| WP_BRAINDERIVED_NEUROTROPHIC_FACTOR_BDNF_SIGNALING_PATHWAY | 84 | -0.50568 | -1.94094 | 5.13E-05 | 0.008448 | 0.007244 |
| PID_CMYB_PATHWAY | 65 | -0.53289 | -1.9353 | 5.44E-05 | 0.008448 | 0.007244 |
| WP_VEGFAVEGFR2_SIGNALING_PATHWAY | 241 | -0.37673 | -1.68942 | 3.78E-05 | 0.008448 | 0.007244 |
| WP_TCELL_ANTIGEN_RECEPTOR_TCR_PATHWAY_DURING_STAPHYLOCOCCUS_AUREUS_INFECTION | 44 | -0.58755 | -1.96836 | 8.9E-05 | 0.012831 | 0.011003 |
| REACTOME_METABOLISM_OF_POLYAMINES | 38 | 0.577809 | 1.982774 | 0.000163 | 0.01933 | 0.016577 |
| WP_IL4_SIGNALING_PATHWAY | 37 | -0.53843 | -1.75048 | 0.002467 | 0.075773 | 0.064979 |
| WP_MAPK_SIGNALING_PATHWAY | 159 | -0.3584 | -1.52487 | 0.003019 | 0.077637 | 0.066577 |
| BIOCARTA_IL6_PATHWAY | 15 | -0.7087 | -1.87039 | 0.003375 | 0.078734 | 0.067518 |
| WP_IL3_SIGNALING_PATHWAY | 35 | -0.52524 | -1.68502 | 0.004629 | 0.08586 | 0.073629 |
| PID_MAPK_TRK_PATHWAY | 20 | -0.61758 | -1.78418 | 0.005831 | 0.094894 | 0.081375 |

GSEA，Gene Set Enrichment Analysis.
